# (*R*)-2-Phenyl-4,5-Dihydrothiazole-4-Carboxamide Derivatives Containing a Diacylhydrazine Group: Synthesis, Biological Evaluation, and SARs

**DOI:** 10.3390/molecules24244440

**Published:** 2019-12-04

**Authors:** Feng-Yun Li, Jing-Bo Liu, Jia-Ning Gong, Gen Li

**Affiliations:** 1 College of Chinese Materia Medica, Tianjin University of Traditional Chinese Medicine, Tianjin 300193, Chinagongjianing991208@163.com (J.-N.G.); 2 College of Horticulture and Landscape Architecture, Tianjin Agricultural University, Tianjin 300384, China; liujingbo0626@163.com

**Keywords:** (R)-2-Phenyl-4,5-dihydrothiazole-4-carboxamide, diacylhydrazine, biological activity, CoMSIA model, insecticidal mechanism

## Abstract

A series of (R)-2-phenyl-4,5-dihydrothiazole-4-carboxamide derivatives containing a diacylhydrazine moiety were designed and synthesized. Their structures were confirmed by melting points, ^1^H NMR, ^13^C NMR, and elemental analysis (EA). Their antifungal and insecticidal activities were evaluated. The antifungal activity result indicated that most title compounds against *Cercospora arachidicola*, *Alternaria solani*, *Phytophthora capsici*, and *Physalospora piricola* exhibited apparent antifungal activities at 50 mg/L, and better than chlorothalonil or carbendazim. The EC_50_ values of (*R*)-*N*’-benzoyl-2-(4-chlorophenyl)-4,5-dihydrothiazole-4-carbohydrazide (**I-5**) against six tested phytopathogenic fungi were comparable to those of chlorothalonil. The CoMSIA model showed that a proper hydrophilic group in the R^1^ position, as well as a proper hydrophilic and electron-donating group in the R^2^ position, could improve the antifungal activity against *Physalospora piricola*, which contributed to the further optimization of the structures. Meanwhile, most title compounds displayed good insecticidal activities, especially compound (*R*)-*N*’-(4-nitrobenzoyl)-2-(4-nitrophenyl)-4,5-dihydrothiazole-4-carbohydrazide (**III-3**). The insecticidal mechanism results indicated that compound **III-3** can serve as effective insect Ca^2+^ level modulators by disrupting the cellular calcium homeostasis in *Mythimna separata*.

## 1. Introduction

By 2050, the global population is projected to grow to over 9 billion, with the associated demand for increasing food production [1,2]. Plant diseases mainly caused by fungi, viruses, oomycetes, and bacteria have caused severe losses to the yield of crops in the world per year. The control of plant diseases in agriculture is very important. Some efficient measures have been taken to resolve the above problem, and it is well known that the invention and use of agrochemicals play an important role in reducing crop loss caused by plant diseases. However, many fungal, virus, oomycete, and bacteria species have developed resistance to some of the currently available agrochemicals due to long-term use of traditional pesticides with a single mode of action [3,4,5,6]. Moreover, the studies found that a large-scale outburst of drug resistance can be fast accelerated within a relatively short period once resistant populations emerge, which will further increase the difficulty in solving the severe problem and the potential risks on food production [7,8]. Therefore scientists have been dedicated to developing novel agrochemicals with innovative skeletons, novel mechanisms of action, and eco-friendly characteristics.

In drug design processes, one of the most effective and promising ways to discover biological active structures is based on existing pharmacological skeletons from natural products with various bioactivities [9,10,11]. (R)-2-(2-Hydroxyphenyl)-4,5-dihydrothiazole-4-carboxylic acid, as a natural product representative of 2-aryl-4,5-dihydrothiazole-4-carboxylic acid derivatives, has been confirmed to possess anticancer activity for L1210 cell lines in vitro [12]. In the past years, researchers have reported many 2-aryl-4,5-dihydrothiazole-4-carboxylic acid derivatives with a variety of biological activities in medicinal chemistry, including antibiotic [13], anti-HIV [14], and anticancer activities [15]. However, few researches about the application in plant disease prevention have been reported. Due to low toxicity, good biodegradability, and compatibility, natural products (NPs) have been broadly used as a source of inspiration for many commercial synthetic organic fungicides, herbicides, and insecticides, such as coumoxystrobin [16], glufosinate–ammonium [17], berberine [18,19,20], and rotenone [21,22]. Moreover, natural products have also frequently served as lead compounds in developing novel synthetic pesticides, which possess better efficacies than natural products [23]. Recently, our research group found that some (R)-2-aryl-4,5-dihydrothiazole-4-carboxylic acid derivatives containing amide and ester moieties displayed remarkable and broad-spectrum antifungal activities [24,25], indicating that (R)-2-aryl-4,5-dihydrothiazole-4-carboxylic acid derivatives could be used as new potential lead structures for the development of antifungal agents.

It is well known that the nitrogen of acylhydrazine as an electron-rich group can easily form hydrogen bonds with various enzymes in organisms to display various biological activities, such as fungicidal [26], insecticidal [27,28], herbicidal [29], antivirus [30], antimicrobacterial [31], antitrypanosomal [32], antitumor [33], and antioxidant activities [34]. Introducing acylhydrazine structure into bioactive molecules is a significant method in improving the bioactivities to develop new potent pesticidal candidates with novel modes of action by binding to different target receptors.

**Figure 1:**
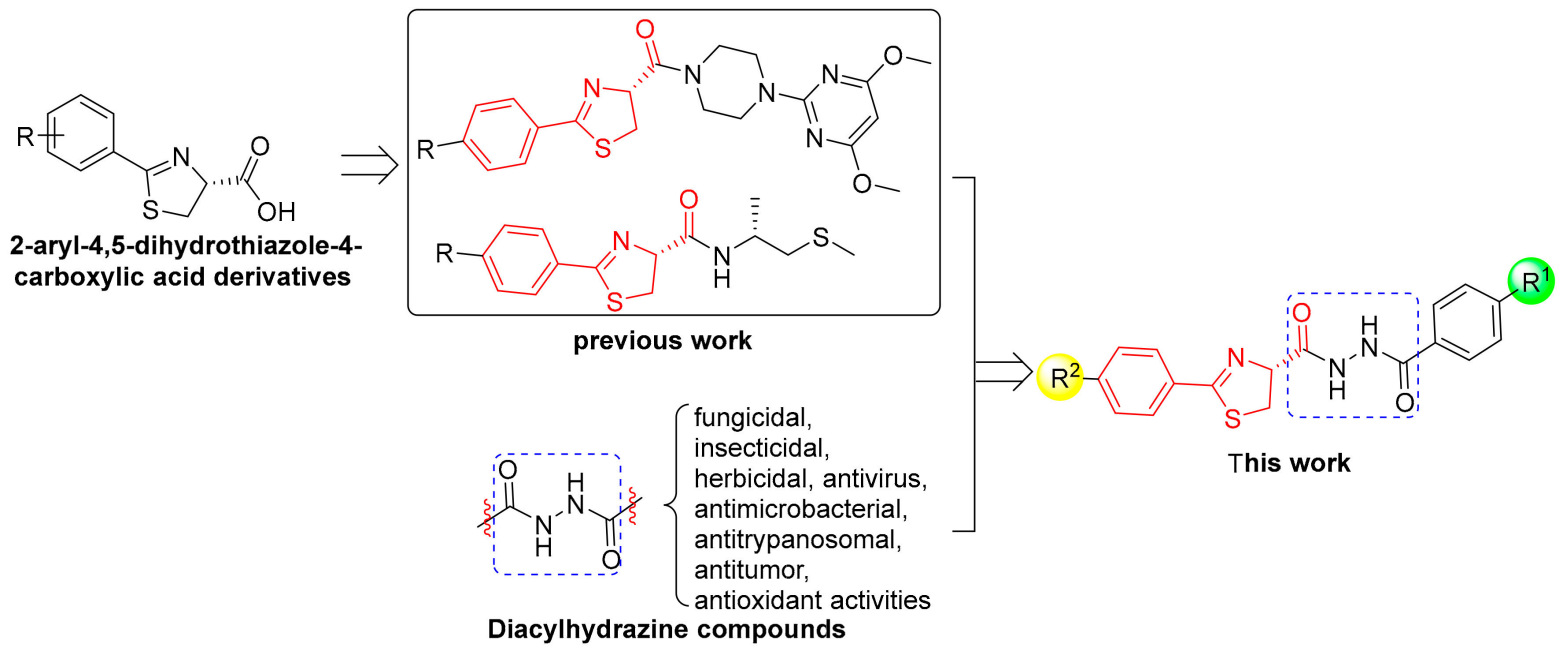
Design strategy of the title compounds.

Inspired by the descriptions above, a series of (R)-2-phenyl-4,5-dihydrothiazole-4-carboxamide derivatives containing a diacylhydrazine moiety (Figure 1) were designed and synthesized, and their structures were confirmed by melting points, ^1^H NMR, ^13^C NMR, and elemental analysis (EA). Their biological activities were investigated accordingly. The preliminary structure activity relationship (SAR) was analyzed as well, and the preliminary insecticidal mechanism was explored with the calcium imaging technique.

## 2. Results and Discussion

### 2.1. Chemistry

**Scheme 1:**
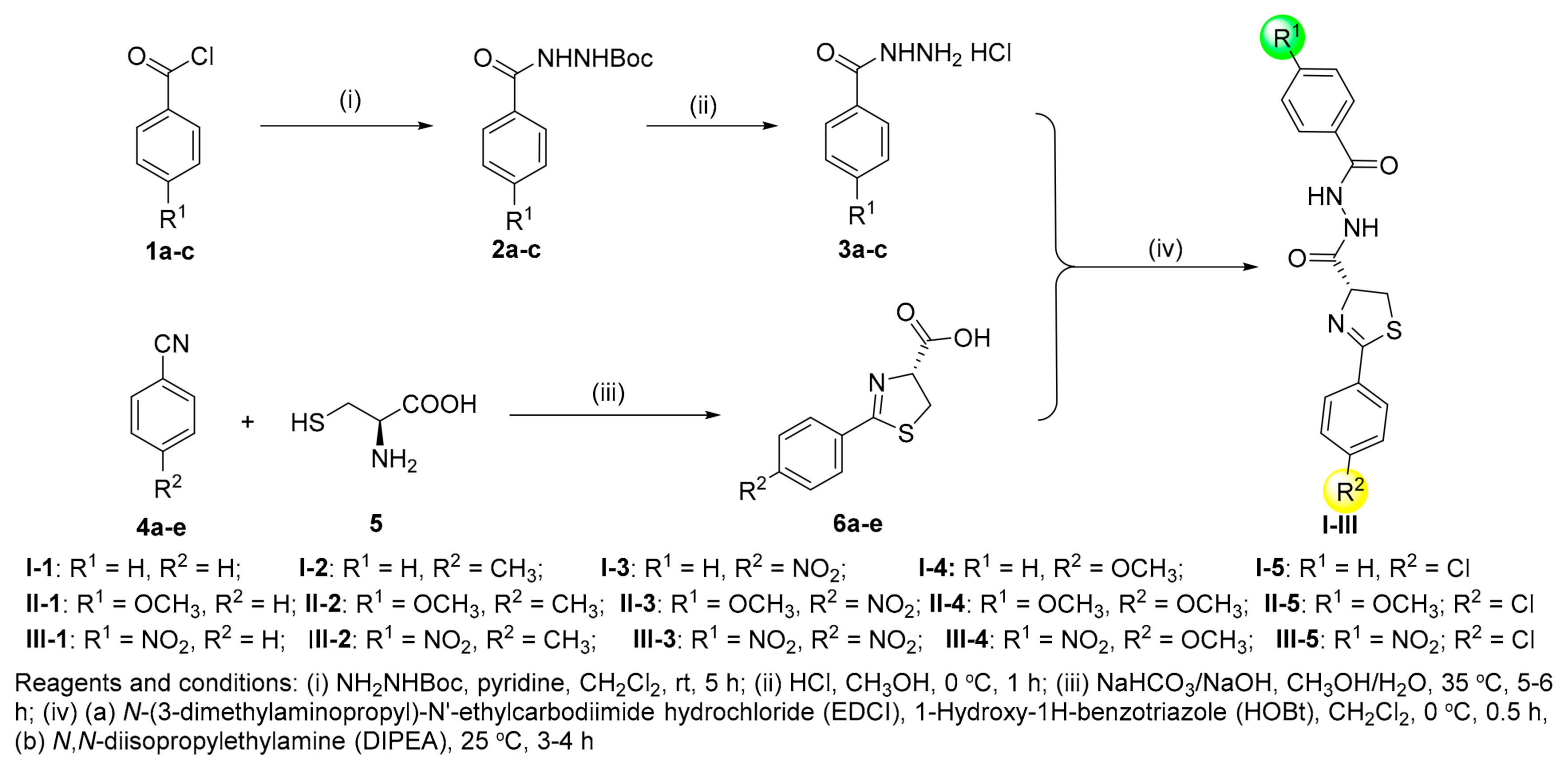
Synthetic route of title compounds **I–III**.

The synthetic routes of the title compounds are shown in Scheme 1. Intermediates **2** were prepared by treated benzoyl chloride derivatives **1** with tert-butylcarbazate [35]. After that, intermediates **3** were obtained by treating intermediates **2** with 4 mol/L hydrochloride in methanol in good yields. Intermediates **6** were synthesized by treating L-cysteine **5** with benzonitrile derivatives **4** in the presence of sodium bicarbonate and sodium hydroxide in the mixture of methanol and water, according to our previous report with some improvement [25]. Initial attempts to synthesize the title compounds **I–III** by coupling intermediates **3** and **6** with *N*-(3-dimethylaminopropyl)-*N*’-ethylcarbodiimide hydrochloride (EDCI) and 1-hydroxy-1*H*-benzotriazole (HOBT) as coupling reagents in the presence of triethylamine (Et_3_N) as acid binding reagent, however, yielded very low title compounds. Then, with the improvement of reaction conditions, *N*,*N*-diisopropylethylamine (DIPEA) was used as the acid binding reagent, and title compounds **I–III** were obtained in moderate yields. The structures of title compounds were confirmed by ^1^H NMR, ^13^C NMR, and elemental analysis (EA).

### 2.2. Biological Activity and Structure Activity Relationships

#### 2.2.1. Antifungal Activity and 3D-QSAR

To explore the relationship between activities of title compounds with different types of functional groups R^1^ and R^2^ on the benzene ring, compounds with R^1^ (H, CH_3_, NO_2_) and R^2^ (H, CH_3_, NO_2_, OCH_3_, Cl) were designed and synthesized. Title compounds **I–III** against six phytopathogenic fungi were evaluated by the mycelium growth rate test according to the literature [36]. As shown in Table 1, most of the compounds against *Cercospora arachidicola*, *Alternaria solani*, *Phytophthora capsici*, and *Physalospora piricola* displayed obvious antifungal activities (in vitro) at the concentration of 50 mg/L, and better than the control chlorothalonil or carbendazim. Furthermore, it is worthy to note that compounds **I-1** and **I-5** exhibited broad-spectrum antifungal activities. On the whole, when the group R^1^ was the same, the group R^2^ on the benzene ring exerted different effects on antifungal activity following the order Cl and H > CH_3_ > OCH_3_ and NO_2_. For example, **I-1** (R^2^ = H) and **I-5** (R^2^ = Cl) against *Phytophthora capsici* exhibited 71.5% and 72.4% antifungal activities, respectively, whereas **I-2** (R^2^ = CH_3_), **I-3** (R^2^ = OCH_3_), and **I-4** (R^2^ = NO_2_) displayed 64.2%, 52.7%, and 50.2% antifungal activities, respectively. Meanwhile, when the group R^2^ was the same, the sequence of the antifungal activities of these compounds with R^1^ was H > CH_3_ and NO_2_. From Table 1, the antifungal activities of compounds **I** (R^1^ = H) were better than those of **II** (R^1^ = OCH_3_) and **III** (R^1^ = NO_2_). These results clearly indicate that the groups R^1^ = H and R^2^ = Cl or H on the benzene ring had a positive effect on the antifungal activities. Meanwhile, the antifungal activities of compounds **I-1**, **II-1**, and **III-1** were better than that of **6a**, indicating that introducing diacylhydrazine moiety could improve the antifungal activity. 

The EC_50_ values of compound **I-5** against six phytopathogenic fungi were further tested and are displayed in Table 2. From it, the EC_50_ values of compound **I-5** were comparable to those of chlorothalonil. Moreover, the EC_50_ value of compound **I-5** against *Physalospora piricola* was lower than that of chlorothalonil, indicating that compound **I-5** against *Physalospora piricola* exhibited better antifungal activity compared with chlorothalonil.

**Figure 2:**
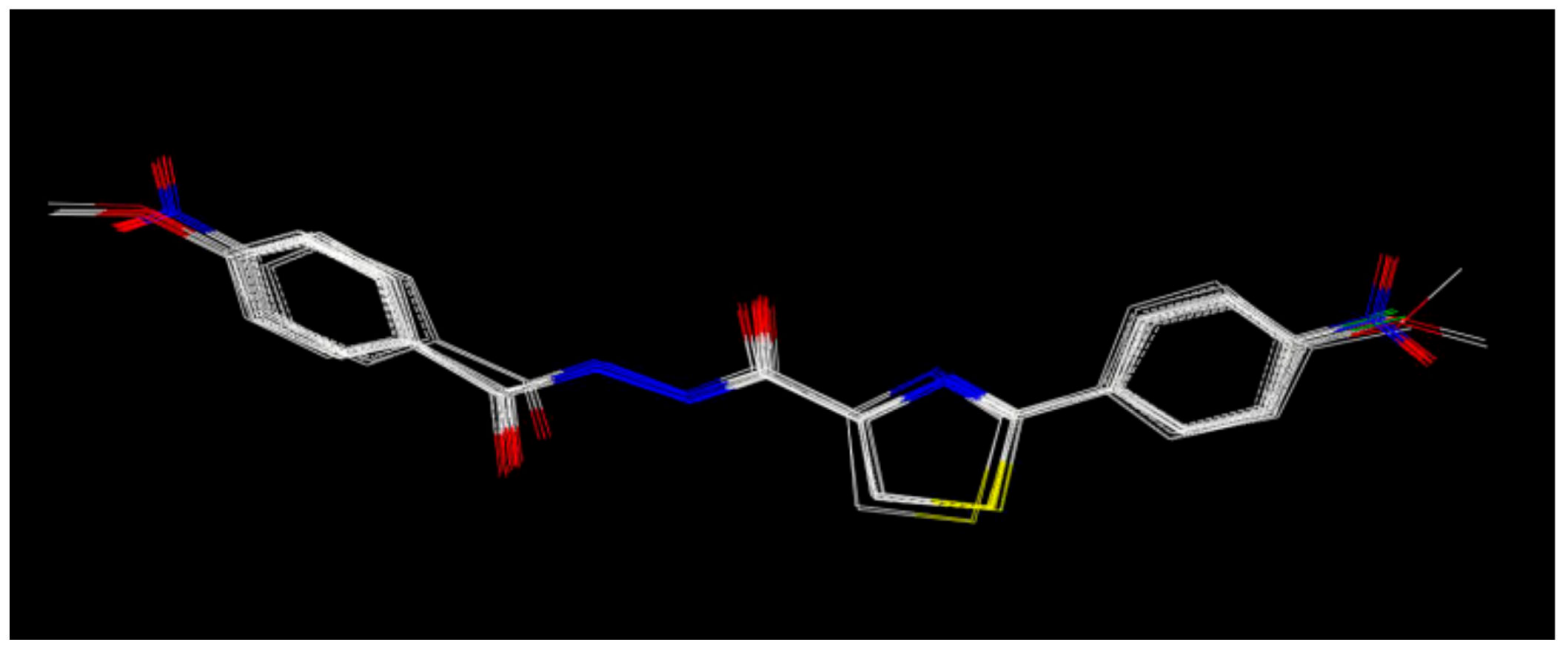
Alignment of compounds **I–III**.

In order to elucidate the substitution effect on antifungal activity against *Physalospora piricola* of the title compounds, a brief 3D-QASR analysis was carried out using Sybyl 6.9 software based on pEC_50_ in Table 3 (Figure 2). The obtained 3D-QASR model was in agreement with the statistically recommended values (*q*^2^ > 0.5, *r*^2^ > 0.8). To better reveal the 3D-QSAR results, **I-5** was used as the template molecule by virtue of the best antifungal activity. Steric, hydrophobic, electrostatic, H-bond donor, and H-bond acceptor fields were used to build the CoMSIA model, and the sequence of the relative contribution of them to the 3D-QASR model was electrostatic (47.5%) > hydrophobic (34.5%) > H-bond acceptor (12.6%) > steric (5.4%) > H-bond donor (0%), which suggests that the antifungal activity was mainly determined by electrostatic and hydrophobic fields.

**Figure 3:**
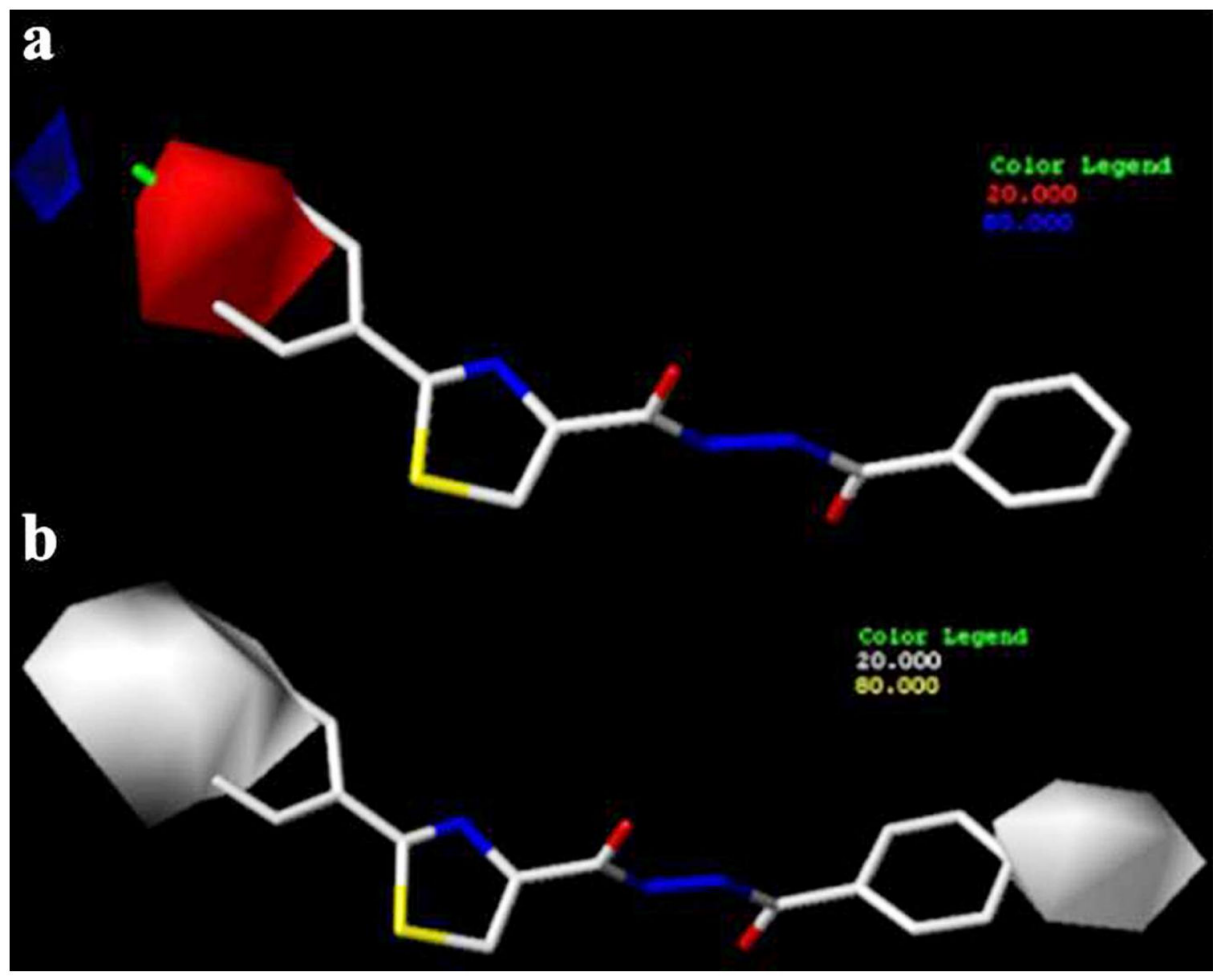
The contour map of the electrostatic field (**a**) and the hydrophobic field (**b**).

The CoMSIA contour maps in Figure 3 show how the electrostatic and hydrophobic fields contribute to the antifungal activities. As for the electrostatic CoMSIA contour map (Figure 3), the red contours are mainly located near the R_2_ group, indicating that placing proper electron-donating groups at this position is favorable to antifungal activities, which is in agreement with the antifungal activities in the sequence of the R^2^ group H and Cl > CH_3_ > OCH_3_ and NO_2_. As shown in the hydrophobic CoMSIA contour map (Figure 3), around the R^1^ and R^2^ groups, the white contours mean that placing proper hydrophilic groups at the positions could improve the antifungal activity, whereas there are almost no yellow contours around the positions, indicating that placing hydrophobic groups is detrimental to activity, which is in agreement with the antifungal activity. The results of the CoMSIA contour maps suggest that a proper hydrophilic group in the R^1^ position, as well as a proper hydrophilic and electron-donating group in the R^2^ position, could improve the antifungal activity against *Physalospora piricola*, which contributes to the further optimization of the structures.

#### 2.2.2. Insecticidal Activity against *Mythimna separata* and Calcium Imaging

The insecticidal activities of title compounds **I**–**III** against *Mythimna separata* were tested and are shown in Table 4. Title compounds **I**–**III** against *Mythimna separata* were determined in the greenhouse according to the reported method [37]. As shown in Table 4, most of them confirm good insecticidal activities at the concentration of 50 mg/L. Especially, compound **III-3** (R^1^ = NO_2_, R^2^ = NO_2_) showed more effective insecticidal activity than those of other compounds at 10 mg/L, which reveals that the groups R^1^ = NO_2_ and R^2^ = NO_2_ on the benzene ring play an important role in the insecticidal activities.

**Figure 4:**
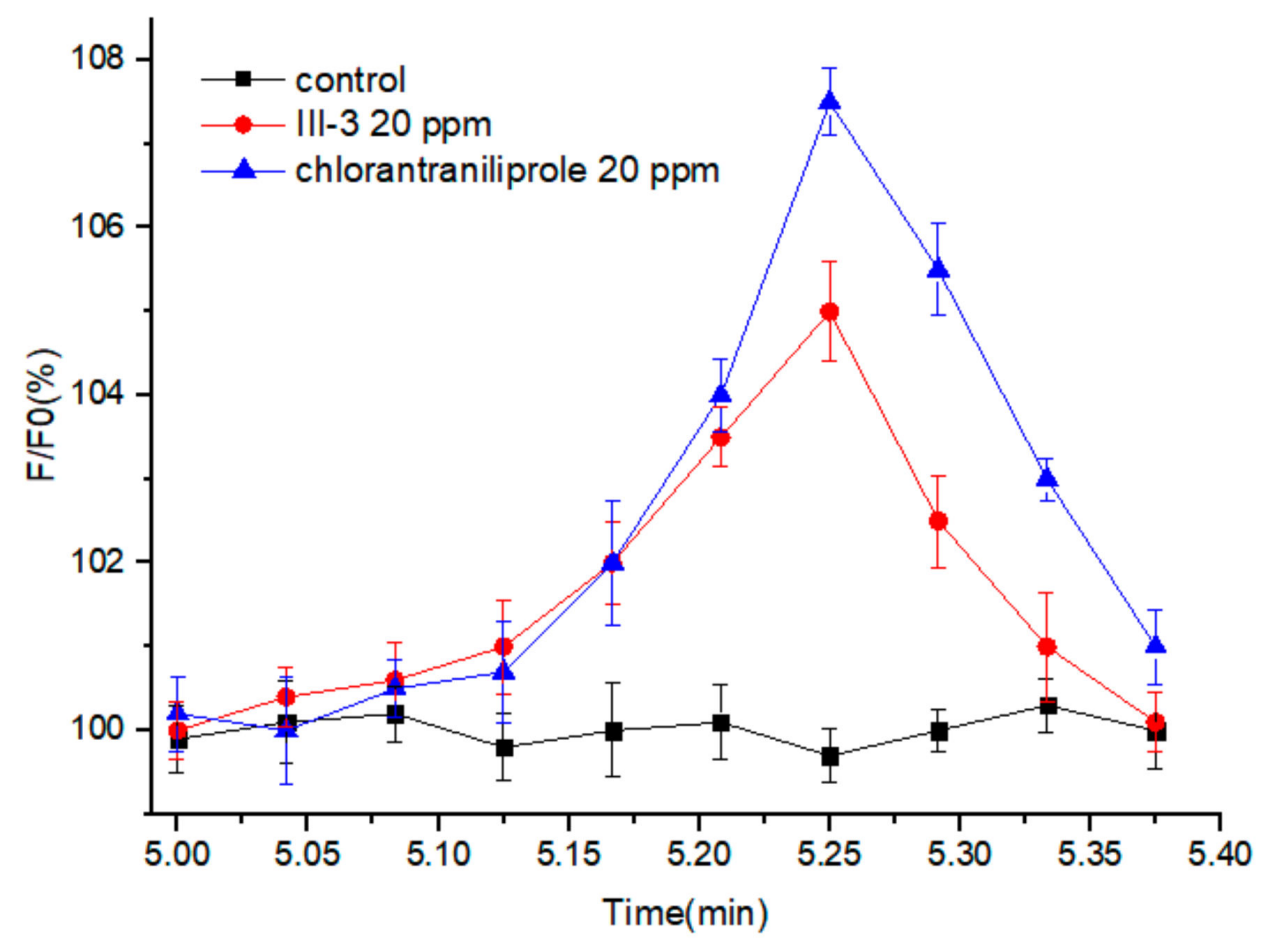
The change of [Ca^2+^]i versus recording time when the central neurons treated with 20 ppm **III-3** and chlorantraniliprole in the absence of extracellular calcium.

To explore the insecticidal mechanism of target compounds, influences of **III-3** on calcium channels in the central neurons isolated from the third instar of *Mythimna separata* were studied with the calcium imaging technique after neuron loading with fluo-3 AM. As shown in Figure 4, compared with the initial value when the central neurons treated with 20 ppm **III-3** and chlorantraniliprole, the peaks of [Ca^2+^]i increased to some extent (**III-3**: 105.2 ± 0.65, *n* = 7; chlorantraniliprole: 107.5 ± 0.78, *n* = 7), respectively. The results indicate that **III-3** can release stored calcium ions, likely from the endoplasmic reticulum (ER) of *Mythimna* *separata* neurons. Combined with our previous work, ryanodine receptors could be the biochemical target of **III-3**, and further research will be needed.

## 3. Materials and Methods

### 3.1. Materials

^1^H NMR and ^13^C NMR were recorded on a Bruker AV400 spectrometer (400 MHz) using CDCl_3_ or DMSO-*d*_6_ as solvent. Chemical shift values (δ) were reported in parts per million (ppm) with tetramethylsilane (TMS) as the internal standard. Melting points were determined on an X-4 binocular microscope melting point apparatus (Beijing Tech Instruments Co., Beijing, China) and uncorrected. Elemental analyses were performed on a Vario EL elemental analyzer (Elementar Co., Germany). Column chromatography purification was carried out using silica gel (200–300 mesh). Reagents were all analytically or chemically pure. All solvents and liquid reagents were dried by standard methods in advance and distilled before use. Intermediate compounds **3a**–**c** and **6a**–**e** were synthesized in Scheme 1 according to the literature [25,35].

### 3.2. General Synthetic Procedure for Title Compounds **I–III**

To a solution of (R)-2-Aryl-4,5-dihydrothiazole-4-carboxylic acid **6** (0.5 mmol), 1-hydroxy-1H-benzotriazole (HOBt, 0.072 g, 0.53 mmol) and *N*-(3-dimethylaminopropyl)-*N*’-ethylcarbodiimide hydrochloride (EDCI, 0.115 g, 0.6 mmol) were subsequently added to dry dichloromethane (10 mL) at 0 °C. After stirring at room temperature for 0.5 h, benzohydrazide hydrochloride **3** (0.5 mmol) and *N*,*N*-diisopropylethylamine (DIPEA) (0.142 g, 1.1 mmol) were subsequently added at 0 °C. The reaction was warmed to room temperature and stirred for 4 h, and then solvent was removed under reduced pressure, and the residue was purified by silica gel column eluted with petroleum ether and ethyl acetate (*V*:*V* = 1:2) to give the title compounds **I–III**. 1H NMR and ^13^C NMR of compounds I-III are available in Supplementary Materials.

*(R)-N’-benzoyl-2-phenyl-4,5-dihydrothiazole-4-carbohydrazide* (**I-1**): White solid, yield 64.3%, m.p. 182–183 °C. ^1^H-NMR (400 MHz, DMSO-*d*_6_) δ 10.52 (s, 1H, CONH), 10.20 (s, 1H, CONH), 7.92 (d, *J* = 7.4 Hz, 2H, Ph-H), 7.90 (d, *J* = 8.3 Hz, 2H, Ph-H), 7.60 (t, *J* = 7.4 Hz, 1H, Ph-H), 7.58 (t, *J* = 7.7 Hz, 1H, Ph-H), 7.54 (t, *J* = 7.5 Hz, 2H, Ph-H), 7.52 (t, *J* = 7.9 Hz, 2H, Ph-H), 5.40 (t, *J* = 9.1 Hz, 1H, CH), 3.85–3.78 (m, 1H, 1/2CH_2_), 3.67–3.62 (m, 1H, 1/2CH_2_). ^13^C-NMR (101 MHz, DMSO-*d*_6_) δ 169.48, 168.57, 165.34, 132.40, 132.29, 131.82, 128.76, 128.47, 128.34, 127.40, 126.54, 78.09, 34.91. Elem. anal. calcd. for C_17_H_15_N_3_O_2_S (%): C, 62.75; H, 4.65; N, 12.91. Found: C, 62.79; H, 4.69; N, 12.93. [*α*]^20^_D_ = +16.2 (*c* 1, MeOH).

*(R)-N’-benzoyl-2-(p-tolyl)-4,5-dihydrothiazole-4-carbohydrazide* (**I-2**): White solid, yield 66.1%, m.p. 187–188 °C. ^1^H-NMR (400 MHz, DMSO-*d*_6_) δ 10.50 (s, 1H, CONH), 10.16 (s, 1H, CONH), 7.90 (d, *J* = 7.4 Hz, 2H, Ph-H), 7.79 (d, *J* = 8.1 Hz, 2H, Ph-H), 7.59 (t, *J* = 7.3 Hz, 1H, Ph-H), 7.51 (t, *J* = 7.5 Hz, 2H, Ph-H), 7.33 (d, *J* = 8.0 Hz, 2H, Ph-H), 5.36 (t, *J* = 9.2 Hz, 1H, CH), 3.79–3.74 (m, 1H, 1/2CH_2_), 3.63–3.58 (m, 1H, 1/2CH_2_). ^13^C-NMR (101 MHz, DMSO-*d*_6_) δ 169.58, 168.36, 165.35, 141.87, 132.38, 131.84, 129.69, 129.29, 128.47, 128.33, 127.39, 78.02, 34.81, 21.03. Elem. anal. calcd. for C_18_H_17_N_3_O_2_S (%): C, 63.70; H, 5.05; N, 12.38. Found: C, 63.75; H, 5.08; N, 12.42. [*α*]^20^_D_ = +14.3 (*c* 1, MeOH).

*(R)-N’-benzoyl-2-(4-nitrophenyl)-4,5-dihydrothiazole-4-carbohydrazide* (**I-3**): White solid, yield 60.2%, m.p. 190–191 °C. ^1^H-NMR (400 MHz, DMSO-*d*_6_) δ 10.51 (s, 1H, CONH), 10.19 (s, 1H, CONH), 7.94–7.89 (m, 4H, Ph-H), 7.62–7.58 (m, 3H, Ph-H), 7.54–7.50 (m, 2H, Ph-H), 5.41 (t, *J* = 9.2 Hz, 1H, CH), 3.85–3.80 (m, 1H, 1/2CH_2_), 3.69–3.64 (m, 1H, 1/2CH_2_). ^13^C-NMR (101 MHz, DMSO-*d*_6_) δ 169.38, 167.50, 165.35, 153.52, 132.37, 131.85, 131.08, 130.09, 128.88, 128.48, 127.39, 78.09, 35.25. Elem. anal. calcd. for C_17_H_14_N_4_O_4_S (%): C, 55.13; H, 3.81; N, 15.13. Found: C, 55.15; H, 3.86; N, 15.14. [*α*]^20^_D_ = +19.8 (*c* 2, MeOH).

*(R)-N’-benzoyl-2-(4-methoxyphenyl)-4,5-dihydrothiazole-4-carbohydrazide* (**I-4**): White solid, yield 62.6%, m.p. 180–181 °C. ^1^H-NMR (400 MHz, DMSO-*d*_6_) δ 10.50 (s, 1H, CONH), 10.15 (s, 1H, CONH), 7.91 (d, *J* = 7.4 Hz, 2H, Ph-H), 7.85 (d, *J* = 8.2 Hz, 2H, Ph-H), 7.60 (t, *J* = 6.9 Hz, 1H, Ph-H), 7.52 (t, *J* = 7.2 Hz, 2H, Ph-H), 7.06 (d, *J* = 8.2 Hz, 2H, Ph-H), 5.34 (t, *J* = 9.0 Hz, 1H, CH), 3.84 (s, 3H, OCH_3_), 3.79–3.74 (m, 1H, 1/2CH_2_), 3.63–3.58 (m, 1H, 1/2CH_2_). ^13^C-NMR (101 MHz, DMSO-*d*_6_) δ 169.69, 167.69, 165.32, 161.95, 132.40, 131.83, 130.17, 128.47, 127.40, 124.96, 114.04, 77.97, 55.43, 34.92. Elem. anal. calcd. for C_18_H_17_N_3_O_3_S (%): C, 60.83; H, 4.82; N, 11.82. Found: C, 60.85; H, 4.84; N, 11.86. [*α*]^20^_D_ = +10.5 (*c* 0.5, MeOH).

*(R)-N’-benzoyl-2-(4-chlorophenyl)-4,5-dihydrothiazole-4-carbohydrazide* (**I-5**): White solid, yield 58.3%, m.p. 176–177 °C. ^1^H-NMR (400 MHz, DMSO-*d*_6_) δ 10.49 (s, 1H, CONH), 10.16 (s, 1H, CONH), 7.89 (d, *J* = 7.3 Hz, 2H, Ph-H), 7.84 (d, *J* = 7.4 Hz, 2H, Ph-H), 7.73 (d, *J* = 7.8 Hz, 2H, Ph-H), 7.58 (t, *J* = 6.7 Hz, 1H, Ph-H), 7.53–7.49 (m, 2H, Ph-H), 5.39 (t, *J* = 9.1 Hz, 1H, CH), 3.84–3.79 (m, 1H, 1/2CH_2_), 3.66–3.63 (m, 1H, 1/2CH_2_). ^13^C-NMR (101 MHz, DMSO-*d*_6_) δ 169.83, 168.13, 165.82, 132.88, 132.33, 132.30, 131.92, 130.74, 128.97, 127.89, 125.95, 78.61, 35.73. Elem. anal. calcd. for C_17_H_14_ClN_3_O_2_S (%): C, 56.74; H, 3.92; N, 11.68. Found: C, 56.77; H, 3.95; N, 11.65. [*α*]^20^_D_ = +13.7 (*c* 1, MeOH).

*(R)-N’-(4-methoxybenzoyl)-2-phenyl-4,5-dihydrothiazole-4-carbohydrazide* (**II-1**): White solid, yield 62.1%, m.p. 197–198 °C. ^1^H-NMR (400 MHz, DMSO-*d*_6_) δ 10.34 (s, 1H, CONH), 10.10 (s, 1H, CONH), 7.89–7.86 (m, 4H, Ph-H), 7.57 (t, *J* = 6.8 Hz, 1H, Ph-H), 7.51 (t, *J* = 7.2 Hz, 2H, Ph-H), 7.03 (d, *J* = 7.6 Hz, 2H, Ph-H), 5.36 (t, *J* = 9.0 Hz, 1H, CH), 3.82 (s, 3H, OCH_3_), 3.80–3.75 (m, 1H, 1/2CH_2_), 3.64–3.59 (m, 1H, 1/2CH_2_). ^13^C-NMR (101 MHz, DMSO-*d*_6_) δ 170.00, 169.01, 165.32, 162.49, 132.80, 132.29, 129.80, 129.25, 128.83, 125.03, 114.19, 78.59, 55.87, 35.40. Elem. anal. calcd. for C_18_H_17_N_3_O_3_S (%): C, 60.83; H, 4.82; N, 11.82. Found: C, 60.86; H, 4.87; N, 11.85. [*α*]^20^_D_ = +16.9 (*c* 1, MeOH). 

*(R)-N’-(4-methoxybenzoyl)-2-(p-tolyl)-4,5-dihydrothiazole-4-carbohydrazide* (**II-2**): White solid, yield 57.5%, m.p. 213–214 °C. ^1^H-NMR (400 MHz, DMSO-*d*_6_) δ 10.35 (s, 1H, CONH), 10.09 (s, 1H, CONH), 7.88 (d, *J* = 7.4 Hz, 2H, Ph-H), 7.78 (d, *J* = 8.3 Hz, 2H, Ph-H), 7.32 (d, *J* = 8.2 Hz, 2H, Ph-H), 7.04 (d, *J* = 7.3 Hz, 2H, Ph-H), 5.34 (t, *J* = 9.2 Hz, 1H, CH), 3.83 (s, 3H, OCH_3_), 3.79–3.73 (m, 1H, 1/2CH_2_), 3.63–3.58 (m, 1H, 1/2CH_2_), 2.38 (s, 3H, CH_3_). ^13^C-NMR (101 MHz, DMSO-*d*_6_) δ 169.58, 168.27, 164.82, 161.99, 141.84, 129.71, 129.28, 128.32, 124.54, 113.69, 78.03, 55.37, 34.82, 21.03. Elem. anal. calcd. for C_19_H_19_N_3_O_3_S (%): C, 61.77; H, 5.18; N, 11.37. Found: C, 61.82; H, 5.18; N, 11.39. [*α*]^20^_D_ = +14.8 (*c* 1, MeOH).

*(R)-N’-(4-methoxybenzoyl)-2-(4-nitrophenyl)-4,5-dihydrothiazole-4-carbohydrazide* (**II-3**): White solid, yield 60.5%, m.p. 202–203 °C. ^1^H-NMR (400 MHz, DMSO-*d*_6_) δ 10.36 (s, 1H, CONH), 10.17 (s, 1H, CONH), 8.35 (d, *J* = 7.7 Hz, 2H, Ph-H), 8.14 (d, *J* = 7.4 Hz, 2H, Ph-H), 7.87 (d, *J* = 7.6 Hz, 2H, Ph-H), 7.03 (d, *J* = 7.7 Hz, 2H, Ph-H), 5.45 (t, *J* = 9.2 Hz, 1H, CH), 3.89–3.84 (m, 1H, 1/2CH_2_), 3.82 (s, 3H, OCH_3_), 3.73–3.68 (m, 1H, 1/2CH_2_). ^13^C-NMR (101 MHz, DMSO-*d*_6_) δ 169.69, 167.76, 165.46, 162.52, 149.67, 138.07, 130.11, 129.81, 124.91, 124.46, 114.21, 78.73, 55.86, 36.02. Elem. anal. calcd. for C_18_H_16_N_4_O_5_S (%): C, 53.99; H, 4.03; N, 13.99. Found: C, 54.02; H, 4.05; N, 14.03. [*α*]^20^_D_ = +16.7 (*c* 1, MeOH).

*(R)-N’-(4-methoxybenzoyl)-2-(4-methoxyphenyl)-4,5-dihydrothiazole-4-carbohydrazide* (**II-4**): White solid, yield 62.2%, m.p. 197–198 °C. ^1^H-NMR (400 MHz, DMSO-*d*_6_) δ 10.34 (s, 1H, CONH), 10.07 (s, 1H, CONH), 7.88 (d, *J* = 7.6 Hz, 2H, Ph-H), 7.84 (d, *J* = 7.4 Hz, 2H, Ph-H), 7.06 (d, *J* = 7.7 Hz, 2H, Ph-H), 7.04 (d, *J* = 7.3 Hz, 2H, Ph-H), 5.32 (t, *J* = 9.2 Hz, 1H, CH), 3.83 (s, 6H, OCH_3_), 3.77–3.72 (m, 1H, 1/2CH_2_), 3.61–3.56 (m, 1H, 1/2CH_2_). ^13^C-NMR (101 MHz, DMSO-*d*_6_) δ 170.21, 168.12, 165.31, 162.47, 162.43, 130.65, 129.80, 125.45, 125.02, 114.52, 114.18, 78.45, 55.92, 55.86, 35.41. Elem. anal. calcd. for C_19_H_19_N_3_O_4_S (%): C, 59.21; H, 4.97; N, 10.90. Found: C, 59.24; H, 4.99; N, 10.94. [*α*]^20^_D_ = +15.1 (*c* 1, MeOH).

*(R)-N’-(4-methoxybenzoyl)-2-(4-chlorophenyl)-4,5-dihydrothiazole-4-carbohydrazide* (**II-5**): White solid, yield 68.1%, m.p. 203–205 °C. ^1^H-NMR (400 MHz, DMSO-*d*_6_) δ 10.34 (s, 1H, CONH), 10.10 (s, 1H, CONH), 7.86 (d, *J* = 7.3Hz, 2H, Ph-H), 7.83 (d, *J* = 7.7 Hz, 2H, Ph-H), 7.73 (d, *J* = 7.8 Hz, 2H, Ph-H), 7.03 (d, *J* = 7.3 Hz, 2H, Ph-H), 5.36 (t, *J* = 8.9 Hz, 1H, CH), 3.82 (s, 3H, OCH_3_), 3.82–3.77 (m, 1H, 1/2CH_2_), 3.67–3.62 (m, 1H, 1/2CH_2_). ^13^C-NMR (101 MHz, DMSO-*d*_6_) δ 169.94, 168.22, 165.44, 162.50, 132.30, 131.88, 130.73, 129.81, 125.95, 124.93, 114.20, 78.56, 55.86. Elem. anal. calcd. for C_18_H_16_ClN_3_O_3_S (%): C, 55.45; H, 4.14; N, 10.78. Found: C, 55.47; H, 4.17; N, 10.82. [*α*]^20^_D_ = +19.5 (*c* 2, MeOH).

*(R)-N’-(4-nitrobenzoyl)-2-phenyl-4,5-dihydrothiazole-4-carbohydrazide* (**III-1**): White solid, yield 63.4%, m.p. 211–212 °C. ^1^H-NMR (400 MHz, DMSO-*d*_6_) δ 10.48 (s, 1H, CONH), 10.17 (s, 1H, CONH), 7.88 (d, *J* = 7.8 Hz, 2H, Ph-H), 7.83 (d, *J* = 7.4 Hz, 2H, Ph-H), 7.73 (d, *J* = 7.6 Hz, 2H, Ph-H), 7.57 (d, *J* = 7.5 Hz, 1H, Ph-H),7.52–7.48 (m, 2H, Ph-H), 5.38 (t, *J* = 8.9 Hz, 1H, CH), 3.83–3.78 (m, 1H, 1/2CH_2_), 3.67–3.62 (m, 1H, 1/2CH_2_). ^13^C-NMR (101 MHz, DMSO-*d*_6_) δ 169.90, 168.27, 165.95, 132.82, 132.39, 132.31, 131.90, 130.74, 129.00, 127.88, 125.96, 78.57, 35.72. Elem. anal. calcd. for C_17_H_14_N_4_O_4_S (%): C, 55.13; H, 3.81; N, 15.13. Found: C, 55.17; H, 3.84; N, 15.15. [*α*]^20^_D_ = +16.5 (*c* 1, MeOH).

*(R)-N’-(4-nitrobenzoyl)-2-(p-tolyl)-4,5-dihydrothiazole-4-carbohydrazide* (**III-2**): White solid, yield 58.8%, m.p. 216–218 °C. ^1^H-NMR (400 MHz, DMSO-*d*_6_) δ: ^1^H-NMR (400 MHz, DMSO-*d*_6_) δ 10.61 (s, 1H, CONH), 10.20 (s, 1H, CONH), 7.90 (d, *J* = 7.7 Hz, 2H, Ph-H), 7.78 (d, *J* = 7.3 Hz, 2H, Ph-H), 7.60 (d, *J* = 7.8 Hz, 2H, Ph-H), 7.33 (d, *J* = 7.4 Hz, 2H, Ph-H), 5.35 (t, *J* = 8.9 Hz, 1H, CH), 3.79–3.74 (m, 1H, 1/2CH_2_), 3.63–3.58 (m, 1H, 1/2CH_2_), 2.37 (s, 3H, CH_3_). ^13^C-NMR (101 MHz, DMSO-*d*_6_) δ 170.11, 168.99, 164.95, 142.41, 137.22, 131.58, 130.14, 129.82, 129.78, 129.13, 128.81, 78.45, 35.29, 21.51. Elem. anal. calcd. for C_18_H_16_N_4_O_4_S (%): C, 56.24; H, 4.20; N, 14.57. Found: C, 56.27; H, 4.23; N, 14.62. [*α*]^20^_D_ = +11.7 (*c* 1, MeOH).

*(R)-N’-(4-nitrobenzoyl)-2-(4-nitrophenyl)-4,5-dihydrothiazole-4-carbohydrazide* (**III-3**): White solid, yield 62.7%, m.p. 225–226 °C. ^1^H-NMR (400 MHz, DMSO-*d*_6_) δ 10.62 (s, 1H, CONH), 10.28 (s, 1H, CONH), 8.35 (d, *J* = 7.8 Hz, 2H, Ph-H), 8.14 (d, *J* = 7.4 Hz, 2H, Ph-H), 7.79 (d, *J* = 7.7 Hz, 2H, Ph-H), 7.59 (d, *J* = 7.4 Hz, 2H, Ph-H), 5.47 (t, *J* = 9.1 Hz, 1H, CH), 3.90–3.85 (m, 1H, 1/2CH_2_), 3.73–3.68 (m, 1H, 1/2CH_2_). ^13^C-NMR (101 MHz, DMSO-*d*_6_) δ 169.64, 167.85, 164.98, 149.69, 138.06, 137.26, 131.54, 130.11, 129.82, 129.14, 124.46, 78.70, 36.01. Elem. anal. calcd. for C_17_H_13_N_5_O_6_S (%): C, 49.16; H, 3.15; N, 16.86. Found: C, 49.18; H, 3.18; N, 16.89. [*α*]^20^_D_ = +19.3 (*c* 1, MeOH).

*(R)-N’-(4-nitrobenzoyl)-2-(4-methoxyphenyl)-4,5-dihydrothiazole-4-carbohydrazide* (**III-4**): White solid, yield 62.4%, m.p. 201–202 °C. ^1^H-NMR (400 MHz, DMSO-*d*_6_) δ 10.58 (s, 1H, CONH), 10.16 (s, 1H, CONH), 7.90 (d, *J* = 7.8 Hz, 2H, Ph-H), 7.83 (d, *J* = 7.4 Hz, 2H, Ph-H), 7.60 (d, *J* = 7.7 Hz, 2H, Ph-H), 7.05 (d, *J* = 7.4 Hz, 2H, Ph-H), 5.32 (t, *J* = 8.6 Hz, 1H, CH), 3.83 (s, 3H, OCH_3_), 3.77–3.72 (m, 1H, 1/2CH_2_), 3.61–3.56 (m, 1H, 1/2CH_2_). ^13^C-NMR (101 MHz, DMSO-*d*_6_) δ 170.15, 168.21, 164.81, 162.46, 137.18, 131.65, 130.65, 129.82, 129.12, 125.44, 114.54, 78.44, 55.93, 35.39. Elem. anal. calcd. for C_18_H_16_N_4_O_5_S (%): C, 53.99; H, 4.03; N, 13.99. Found: C, 54.03; H, 4.07; N, 14.02. [*α*]^20^_D_ = +12.6 (*c* 1, MeOH). 

*(R)-N’-(4-nitrobenzoyl)-2-(4-chlorophenyl)-4,5-dihydrothiazole-4-carbohydrazide* (**III-5**): White solid, yield 58.1%, m.p. 201–202 °C. ^1^H-NMR (400 MHz, DMSO-*d*_6_) δ 10.58 (s, 1H, CONH), 10.18 (s, 1H, CONH), 7.90 (d, *J* = 7.6 Hz, 2H, Ph-H), 7.83 (d, *J* = 7.4 Hz, 2H, Ph-H), 7.73 (d, *J* = 7.7 Hz, 2H, Ph-H), 7.58 (d, *J* = 7.4 Hz, 2H, Ph-H), 5.38 (t, *J* = 8.9 Hz, 1H, CH), 3.84–3.78 (m, 1H, 1/2CH_2_), 3.67–3.62 (m, 1H, 1/2CH_2_). ^13^C-NMR (101 MHz, DMSO-*d*_6_) δ 169.80, 168.17, 164.83, 137.20, 132.30, 131.91, 131.62, 130.73, 129.81, 129.13, 125.95, 78.58, 35.71. Elem. anal. calcd. for C_17_H_13_ClN_4_O_4_S (%): C, 50.44; H, 3.24; N, 13.84. Found: C, 50.46; H, 3.28; N, 13.87. [*α*]^20^_D_ = +14.7 (*c* 1, MeOH).

### 3.3. Biological Assay

#### 3.3.1. Fungicidal Activity

The fungicidal activity of compounds **I**–**III** was tested with the mycelium growth inhibition in vitro against *Cercospora arachidicola*, *Physalospora piricola*, *Alternaria solani*, *Fusarium graminearum*, *Phytophthora capsici*, and *Sclerotinia sclerotiorum*. Chlorothalonil and carbendazim were used as the controls. The relative inhibition ratio (%) was determined through the mycelium growth rate method [36], and calculated using the formula: Relative inhibition rate (%) = (D1 − D2) / D1 × 100%, D1 and D2 are the average diameter of circle mycelia during the blank assay and test assay, respectively. The fungicidal activities of title compounds **I**–**III** at 50 mg/L are shown in Table 1. The EC_50_ values of title compound **I-5** against the six tested fungi are shown in Table 2. The EC_50_ values of title compounds **I**–**III,** chlorothalonil, and carbendazim against Phytophthora piricola are shown in Table 3.

#### 3.3.2. Insecticidal Activity against *Mythimna separata*

The insecticidal activity of **I**–**III** and chlorantraniliprole were tested by the reported method [37], and the result is shown in Table 4.

#### 3.3.3. D-QSAR Calculation Methods

The CoMSIA studies were carried out using a SYBYL v6.9 software from Tripos Inc (St. Louis, MO, USA). All molecules were built with the SKETCH option in SYBYL under default settings. CoMSIA contour maps were generated with partial least-squares coefficients [38]. The partial least-squares was carried out to establish a linear relationship. Cross-validation was performed by using the “leave-one-out” method to obtain the cross-validated coefficient *q*^2^ and optimal number of components. The non-cross-validated correlation coefficient *r*^2^ and cross-validated coefficient *q*^2^ could estimate the predictive capability and modeling, respectively.

### 3.4. Calcium Imaging Experiment

Effects of **III-3** on calcium channels in the central neurons isolated from the third instar of *Mythimna separata* were studied by calcium imaging techniques as described in the literature [37].

## 4. Conclusions

A series of (*R*)-2-phenyl-4,5-dihydrothiazole-4-carboxamide derivatives containing a diacylhydrazine moiety were designed and synthesized, and their chemical structures were identified by melting points, ^1^H-NMR, ^13^C-NMR, and elemental analysis (EA). All of the title compounds **I–III** were evaluated for antifungal and insecticidal activities. The antifungal activity evaluation revealed that most of them against *Cercospora arachidicola*, *Alternaria solani*, *Phytophthora capsici*, and *Physalospora piricola* at 50 mg/L displayed obvious antifungal activities, and were more efficient than chlorothalonil or carbendazim. Especially, compounds **I-1** and **I-5** against the six tested phytopathogenic fungi exhibited broad-spectrum antifungal activities. Furthermore, the EC_50_ values of compound **I-5** were equivalent to those of chlorothalonil. Compound **I-5** can be used as a novel lead structure for antiphytopathogenic fungus agent development. The structure activity relationship indicated that the groups R^1^ = H and R^2^ = Cl or H on the benzene ring had a positive effect on the antifungal activities. Moreover, the CoMSIA model suggested that a proper hydrophilic group in the R^1^ position, as well as a proper hydrophilic and electron-donating group in the R^2^ position, could improve the antifungal activity against *Physalospora piricola*, which contributed to the further optimization of the structures. The insecticidal activities result showed that most of them displayed remarkable insecticidal activities at 50 mg/L, in particular, compound **III-3**. The calcium imaging experiment results indicated that compound **III-3** can serve as effective insect Ca^2+^ level modulators by disrupting the cellular calcium homeostasis in *Mythimna separata*.

## Figures and Tables

**Table 1:** Antifungal activity of the title compounds **I−III.**

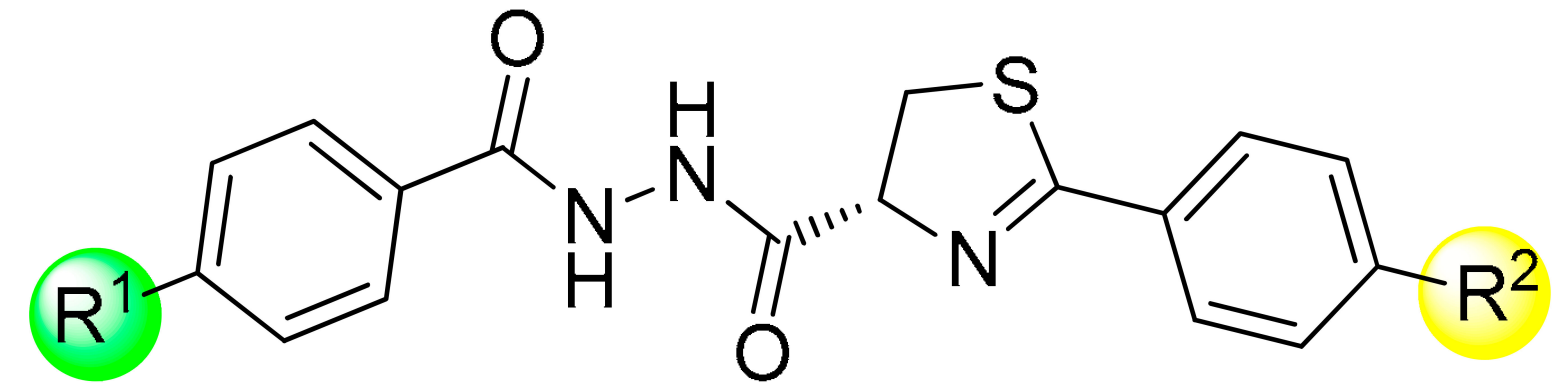								
Compd.	R^1^	R^2^	Antifungal Activity, Inhibition Rate (%, 50 mg/L)					
			*CA* ^1^	*AS*	*PC*	*FG*	*PP*	*SS*
**I-1**	H	H	61.2	56.7	71.5	50.5	75.2	50.7
**I-2**	H	CH_3_	55.1	43.8	64.2	42.4	72.5	46.9
**I-3**	H	NO_2_	48.3	37.3	52.7	35.3	61.9	35.8
**I-4**	H	OCH_3_	50.8	37.4	50.2	35.8	63.2	19.5
**I-5**	H	Cl	64.5	60.4	72.4	52.3	84.3	49.8
**II-1**	OCH_3_	H	48.5	42.2	63.6	35.6	73.8	45.2
**II-2**	OCH_3_	CH_3_	35.7	40.3	57.1	35.2	71.6	30.8
**II-3**	OCH_3_	NO_2_	28.2	34.2	44.3	18.7	54.3	34.8
**II-4**	OCH_3_	OCH_3_	33.5	40.1	43.9	23.6	50.4	13.4
**II-5**	OCH_3_	Cl	47.1	53.4	65.8	36.3	76.7	43.6
**III-1**	NO_2_	H	49.9	55.6	60.5	26.2	67.3	36.7
**III-2**	NO_2_	CH_3_	36.2	50.3	50.3	14.9	63.4	30.2
**III-3**	NO_2_	NO_2_	33.8	30.2	42.6	9.8	51.1	27.7
**III-4**	NO_2_	OCH_3_	36.3	47.3	45.8	13.0	52.5	14.9
**III-5**	NO_2_	Cl	51.6	54.4	60.6	35.7	69.2	33.4
**6a**	--	H	24.8	25.9	33.7	8.3	43.5	10.3
chlorothalonil			60.3	71.5	87.6	40.5	82.5	42.0
carbendazim			12.1	38.3	31.4	99.1	45.6	97.7

^1^*CA: Cercospora arachidicola; AS: Alternaria solani; PC: Phytophthora capsici; FG: Fusarium graminearum; PP: Physalospora piricola; SS: Sclerotinia sclerotiorum*.

**Table 2:** Antifungal activity of the title compounds **I−III.**

Compd.	Fungus	EC_50_	Compd.	Fungus	EC_50_
**I-5**	*Cercospora arachidicola*	15.6	chlorothalonil	*Cercospora arachidicola*	15.5
	*Alternaria solani*	21.5		*Alternaria solani*	17.9
	P*hytophthora capsici*	9.3		*Phytophthora capsici*	5.0
	*Fusarium graminearum*	8.5		*Fusarium graminearum*	8.1
	*Physalospora piricola*	6.1		*Physalospora piricola*	6.6
	*Sclerotinia sclerotiorum*	2.4		*Sclerotinia sclerotiorum*	1.3

**Table 3:** EC_50_ (mg/L) and pEC_50_ of compounds **I–III**, chlorothalonil, and carbendazim against *Phytophthora piricola.*

Compd.	EC_50_	pEC_50_^a^	pEC_50_^b^	Compd.	EC_50_	pEC_50_^a^	pEC_50_^b^
**I-1** ^c^	9.6	2.02	2.13	**II-5**	9.7	2.01	1.97
**I-2**	12.3	1.91	1.94	**III-1**	15.2	1.82	1.87
**I-3** ^c^	16.2	1.79	1.78	**III-2**	16.7	1.78	1.84
**I-4**	19.0	1.72	1.74	**III-3** ^c^	21.2	1.67	1.76
**I-5**	6.1	2.21	2.24	**III-4**	20.5	1.69	1.64
**II-1**	9.8	2.01	1.97	**III-5**	11.3	1.95	2.02
**II-2** ^c^	12.5	1.90	1.99	Chlorothalonil	6.6	-	-
**II-3**	20.1	1.70	1.68	Carbendazim	32.3	-	-
**II-4** ^c^	21.3	1.67	1.60				

^a^ Observed antifungal activity (pEC_50_); ^b^ calculated antifungal activity (pEC_50_); ^c^ the test set molecules.

**Table 4:** Insecticidal activities of title compounds and chlorantraniliprole against *Mythimna separata.*

Compd.	Larvicidal Activity (%) at Concn of (mg/L)			Compd.	Larvicidal Activity (%) at Concn of (mg/L)		
	200 mg/L	50 mg/L	10 mg/L		200 mg/L	50 mg/L	10 mg/L
**I-1**	100	83	50	**II-4**	100	83	23
**I-2**	100	77	43	**II-5**	100	90	53
**I-3**	100	100	67	**III-1**	100	100	53
**I-4**	100	77	33	**III-2**	100	100	57
**I-5**	100	87	57	**III-3**	100	100	**77**
**II-1**	100	83	43	**III-4**	100	87	43
**II-2**	100	100	47	**III-5**	100	77	37
**II-3**	100	100	57	Chlo ^1^	100	100	100

^1^ Chlo: Chlorantraniliprole.

## Supplementary Materials

The following are available online. ^1^H NMR and ^13^C NMR of compounds **I-III** can be accessed online.
