# Supplementary material for: (R)-2-Phenyl-4,5-Dihydrothiazole-4-Carboxamide Derivatives Containing a Diacylhydrazine Group: Synthesis, Biological Evaluation, and SARs
Source: Molecules. 2019 Dec 4;24(24):4440. doi: 10.3390/molecules24244440 (PMC6943579; doi:10.3390/molecules24244440)

Supporting Information

(*R*)-2-Phenyl-4,5-dihydrothiazole-4-carboxamide Derivatives Containing a Diacylhydrazine Group: Synthesis, Biological Evaluation, and SARs

Feng-Yun Li [1], Jing-Bo Liu [2], Jia-Ning Gong [1] and Gen Li [1,*]

[1] College of Chinese Materia Medica, Tianjin University of Traditional Chinese Medicine, Tianjin 300193, China; lifengyun0518@163.com (F.-Y.L); gongjianing991208@163.com (J.-N.G)

[2] College of Horticulture and Landscape Architecture, Tianjin Agricultural University, Tianjin 300384, China; liujingbo0626@163.com

* Correspondence: ligen0725@163.com; Tel.: +00-86-13820392565 (G.L.)

^{1}H NMR and ^{13}C NMR of target compounds I–III

Compound I-1

^{1}H NMR

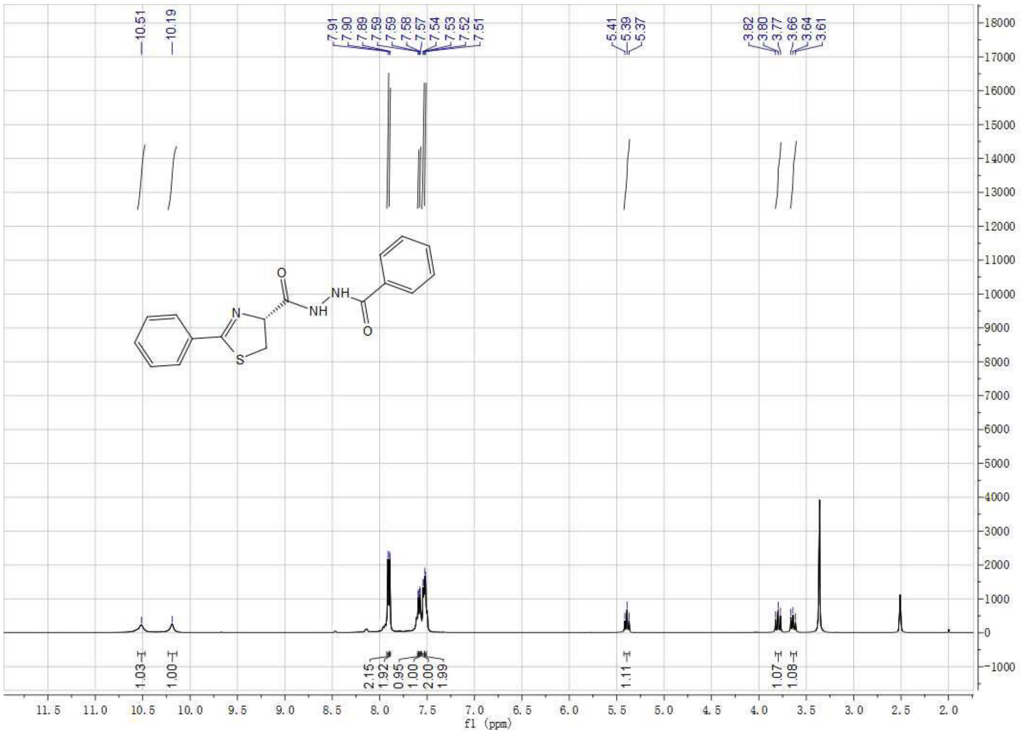

^{13}C NMR

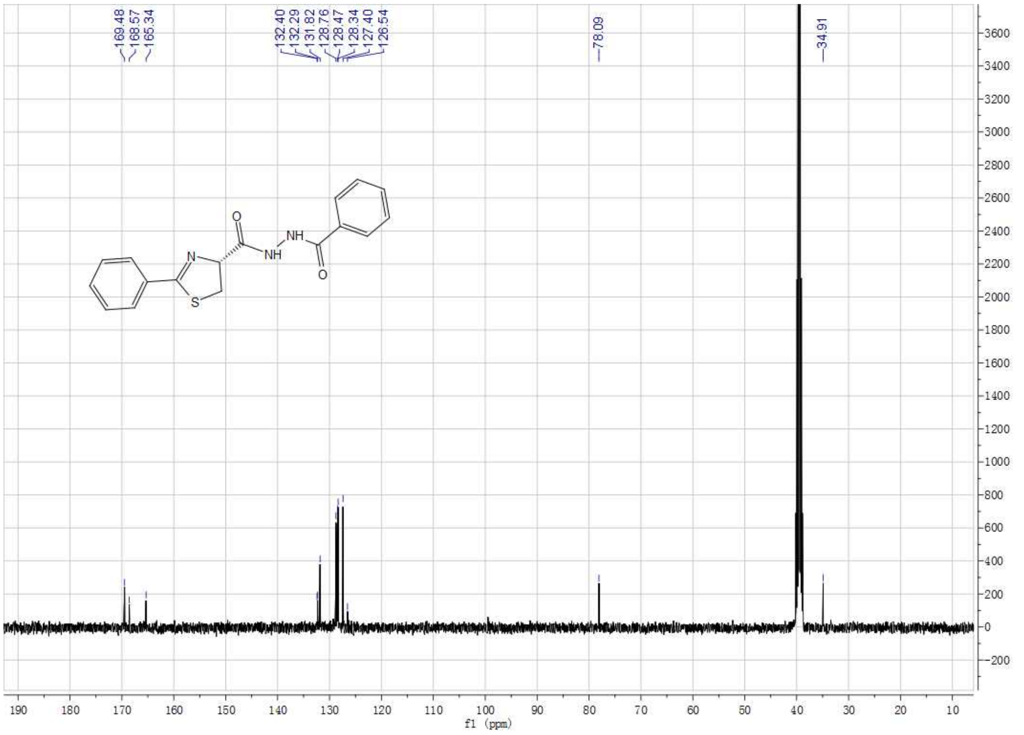

Compound I-2

^{1}H NMR

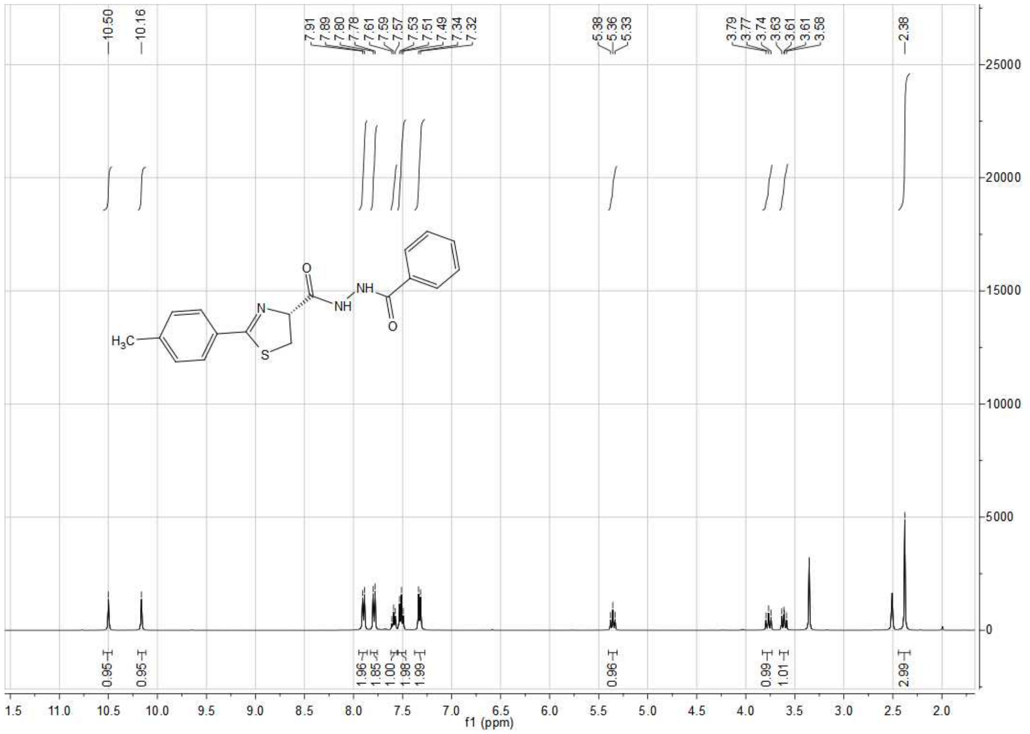

^{13}C NMR

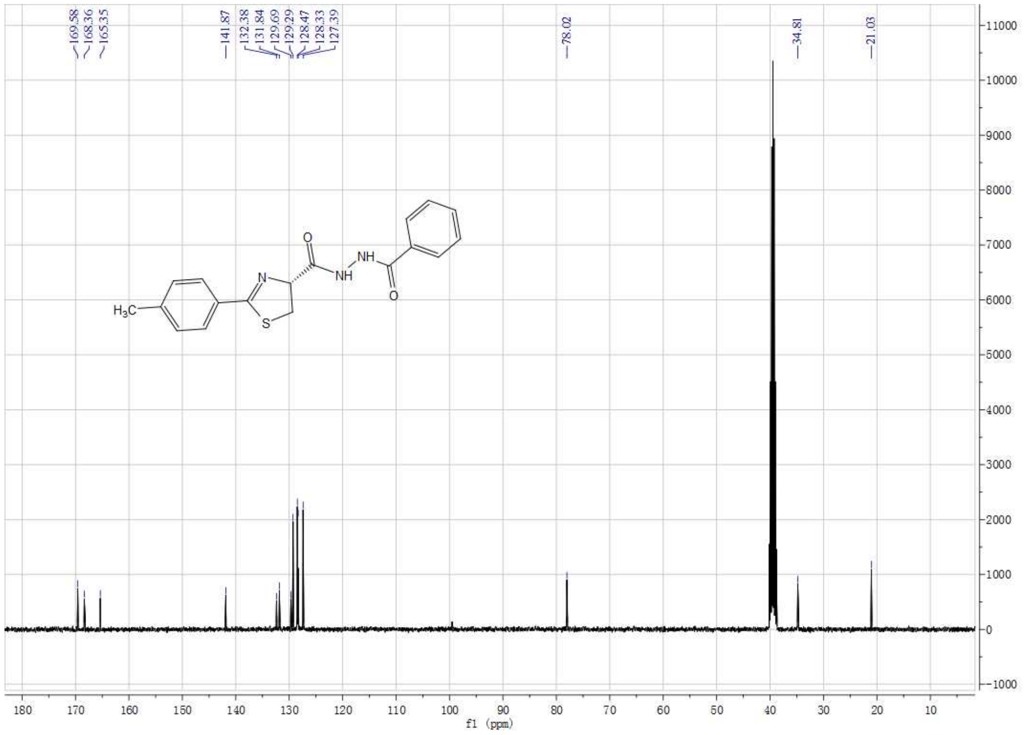

Compound I-3

^{1}H NMR

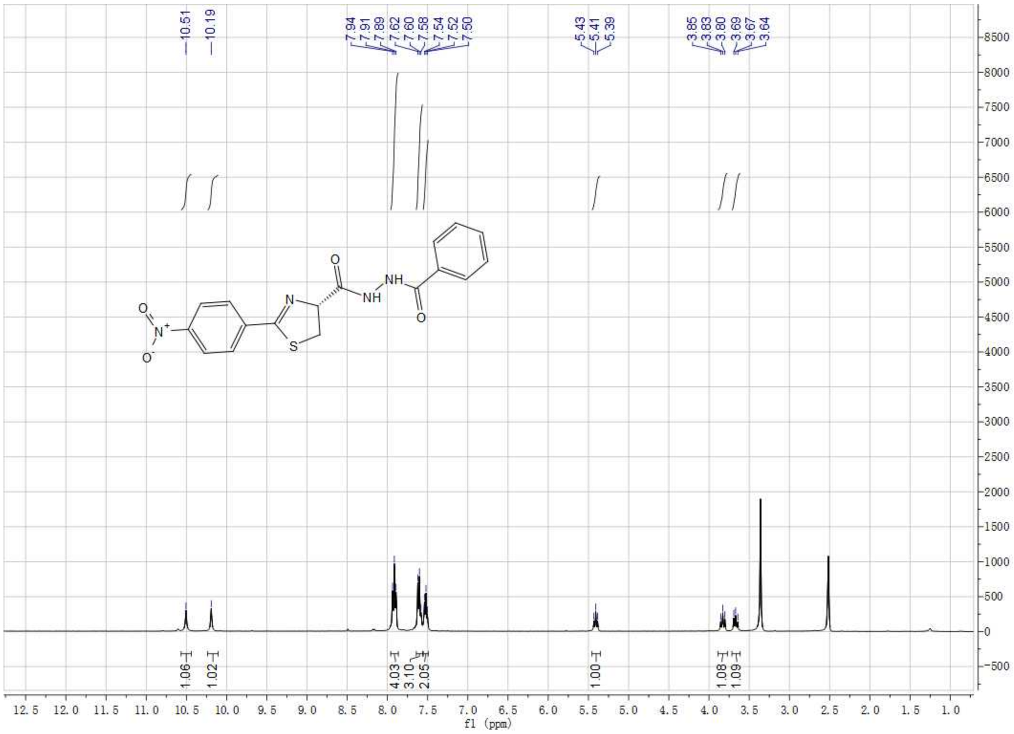

^{13}C NMR

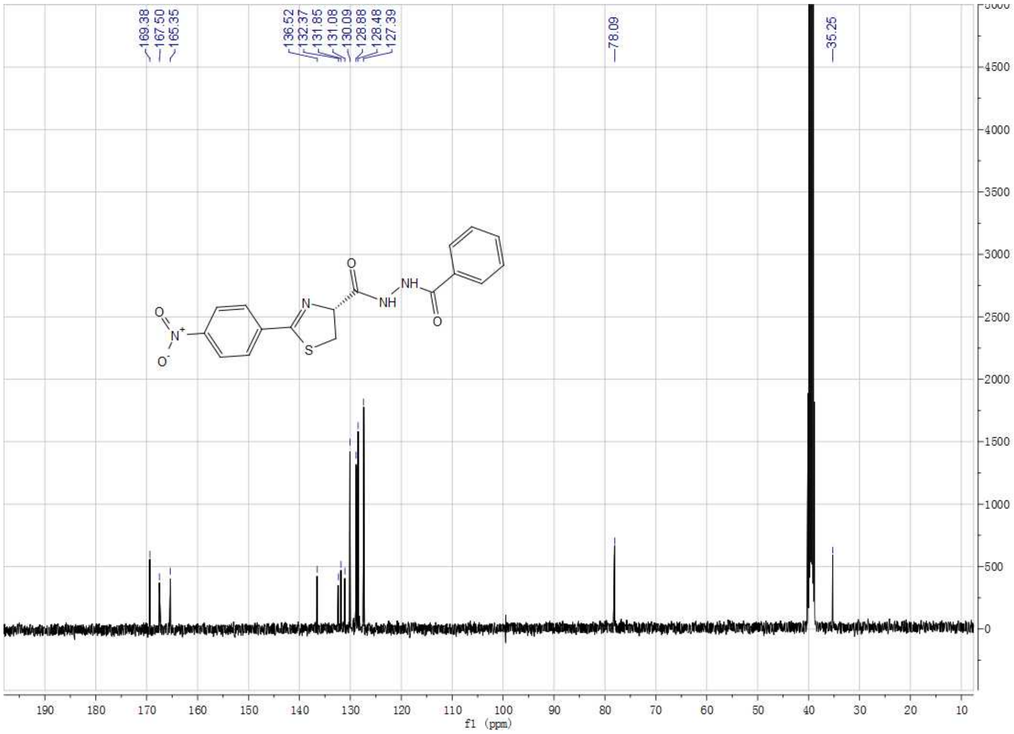

Compound I-4

^{1}H NMR

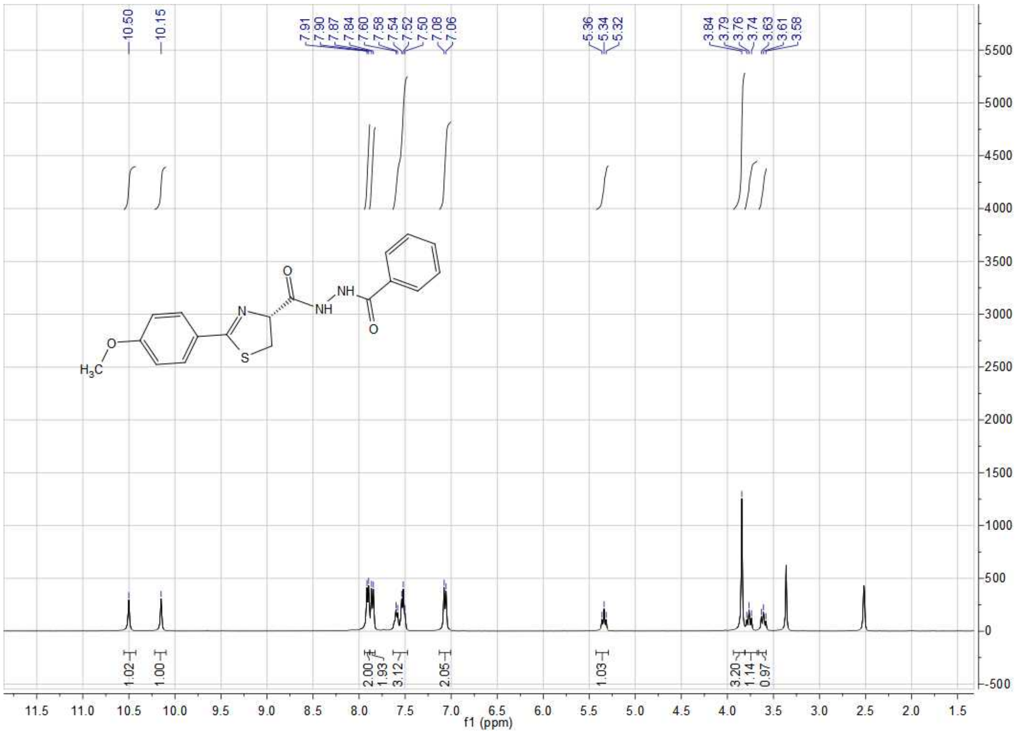

^{13}C NMR

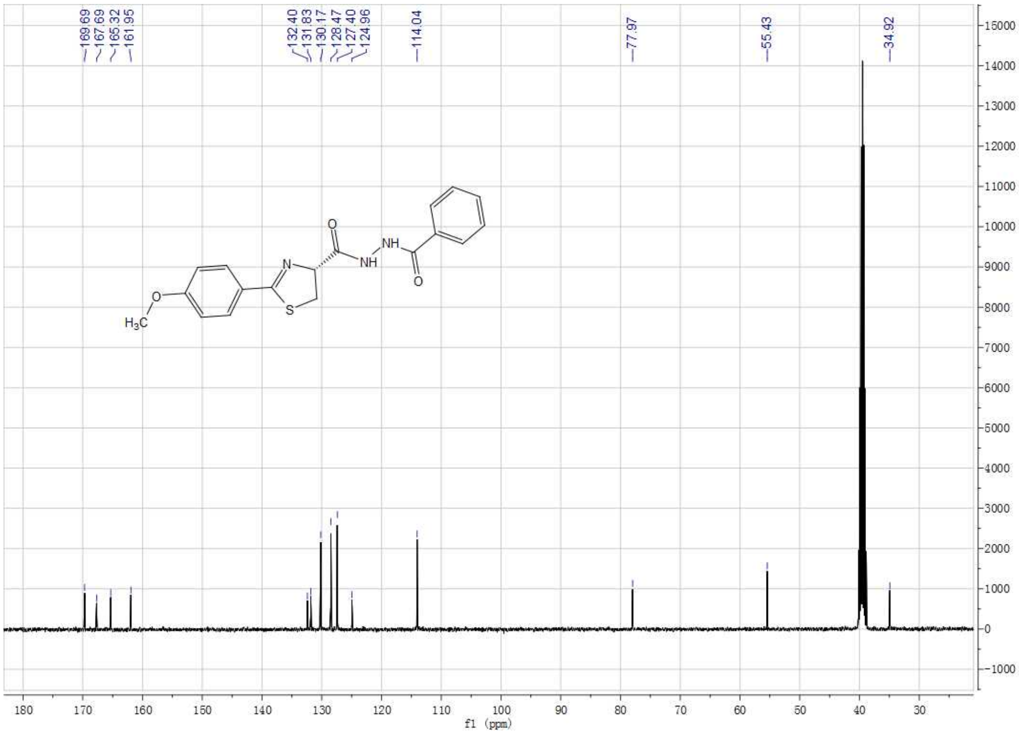

Compound I-5

^{1}H NMR

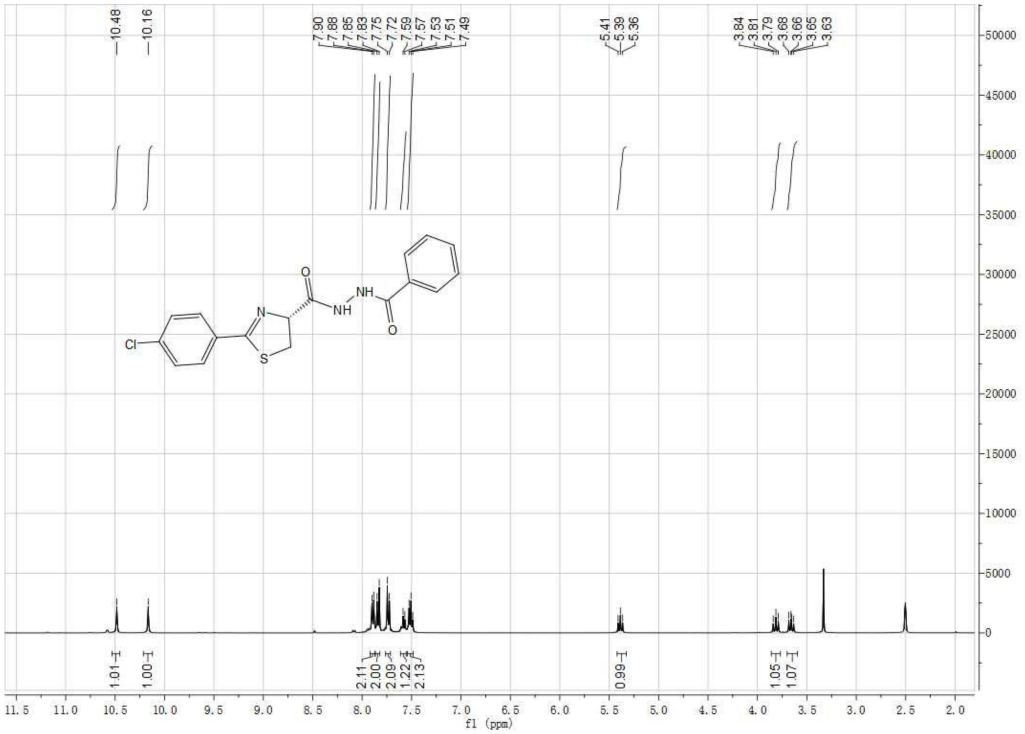

^{13}C NMR

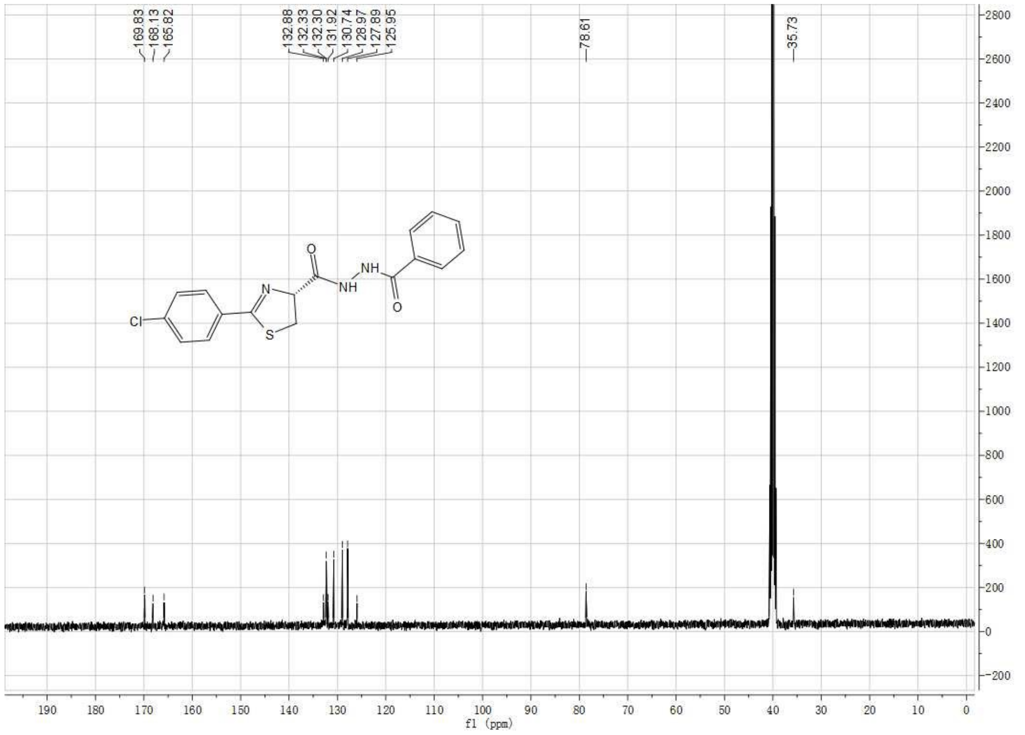

Compound II-1

^{1}H NMR

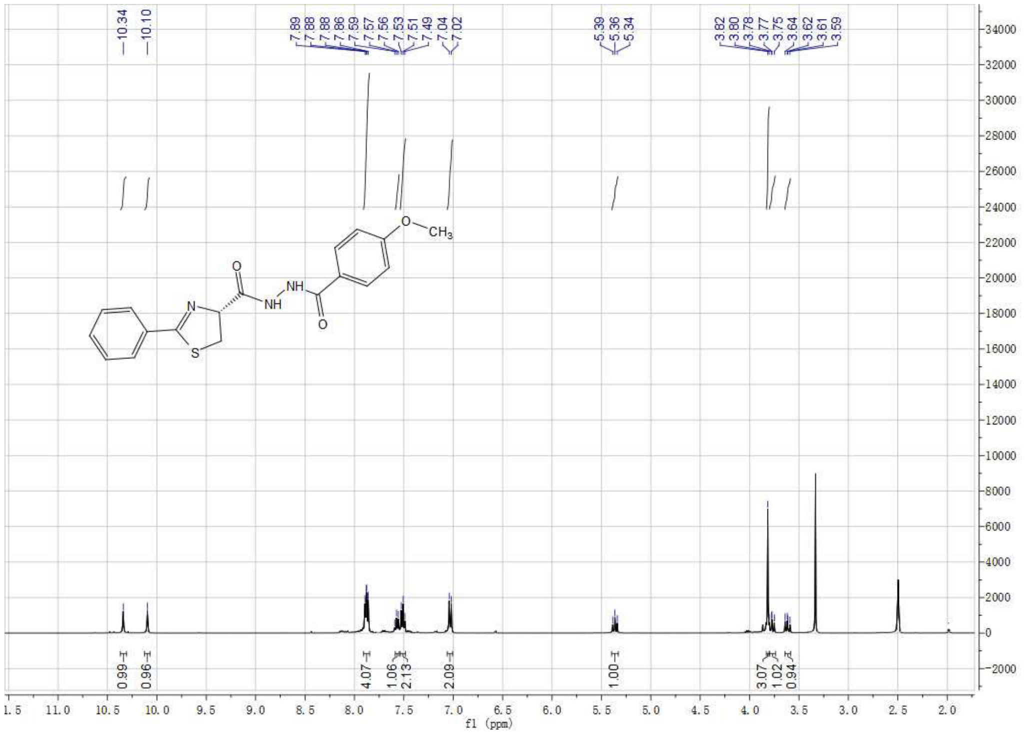

^{13}C NMR

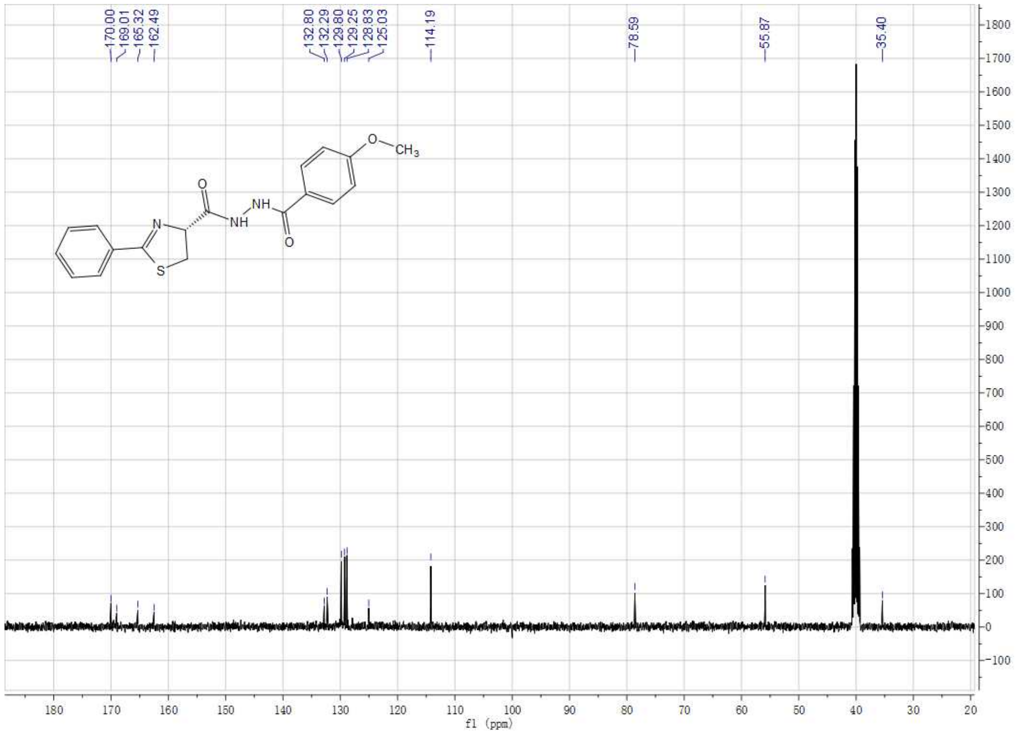

Compound II-2

^{1}H NMR

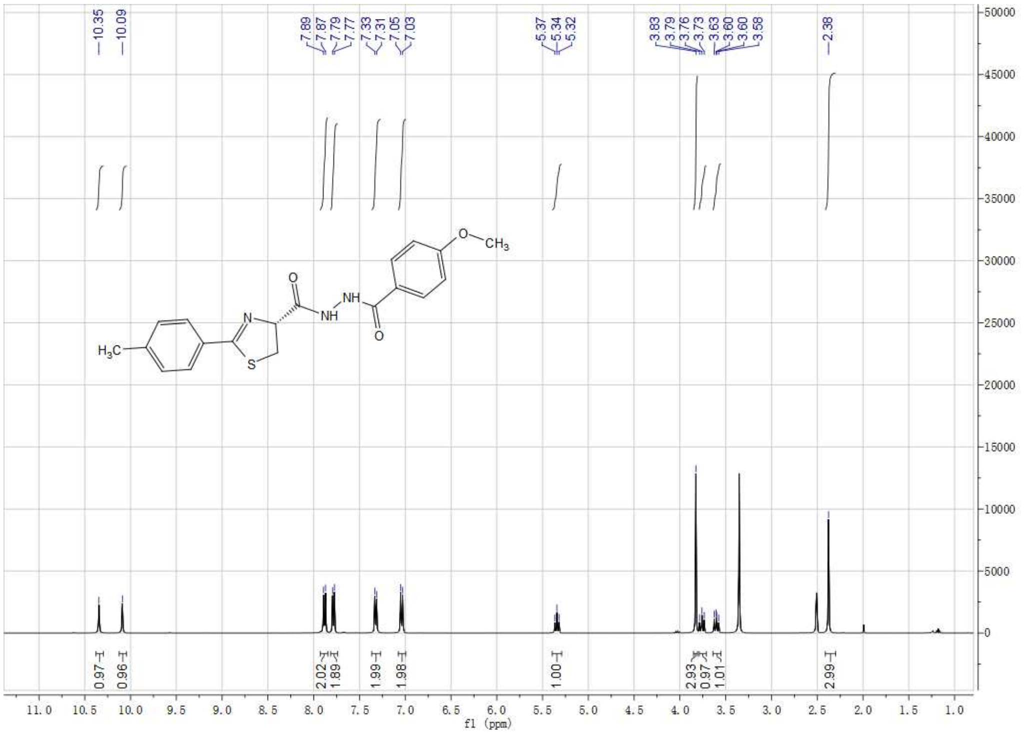

^{13}C NMR

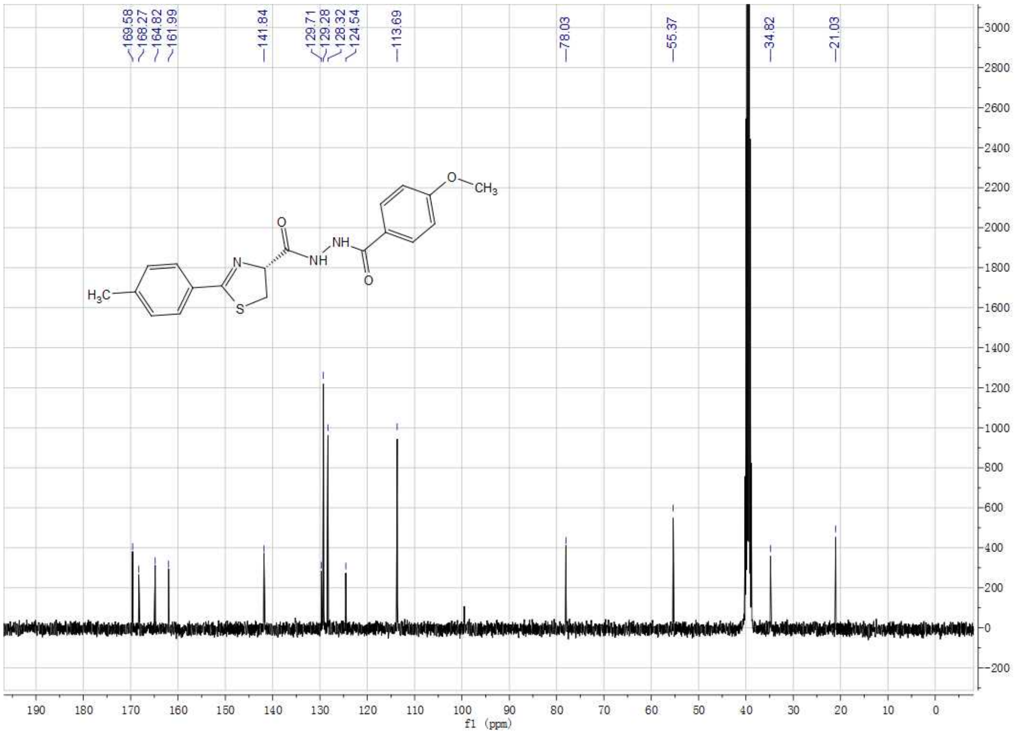

Compound II-3

^{1}H NMR

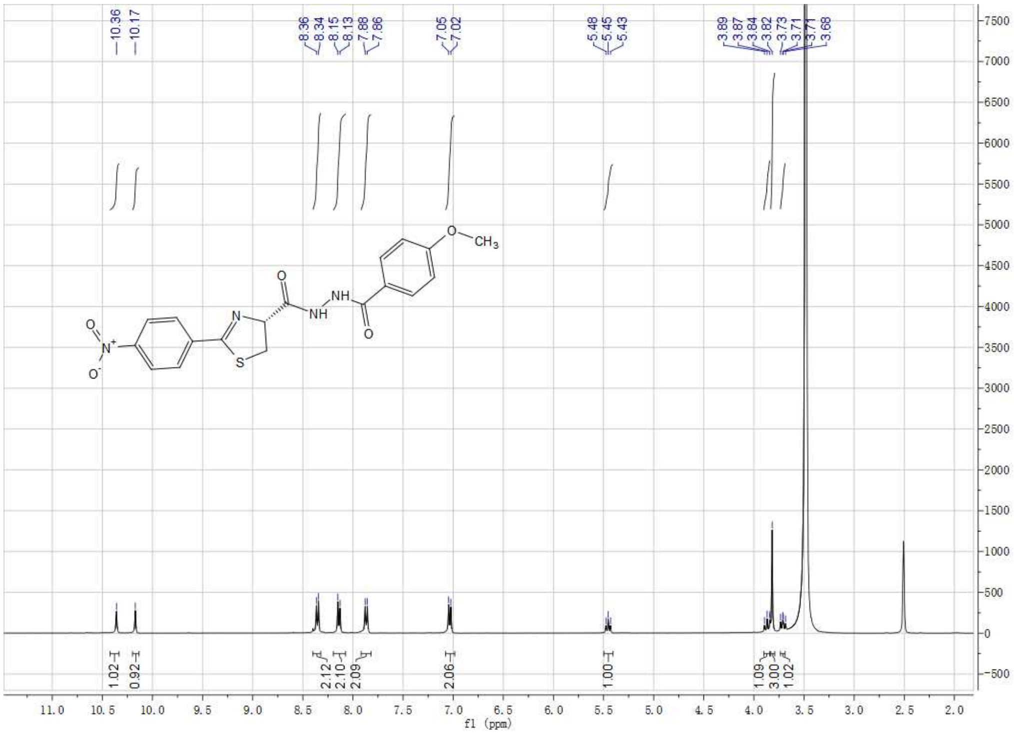

^{13}C NMR

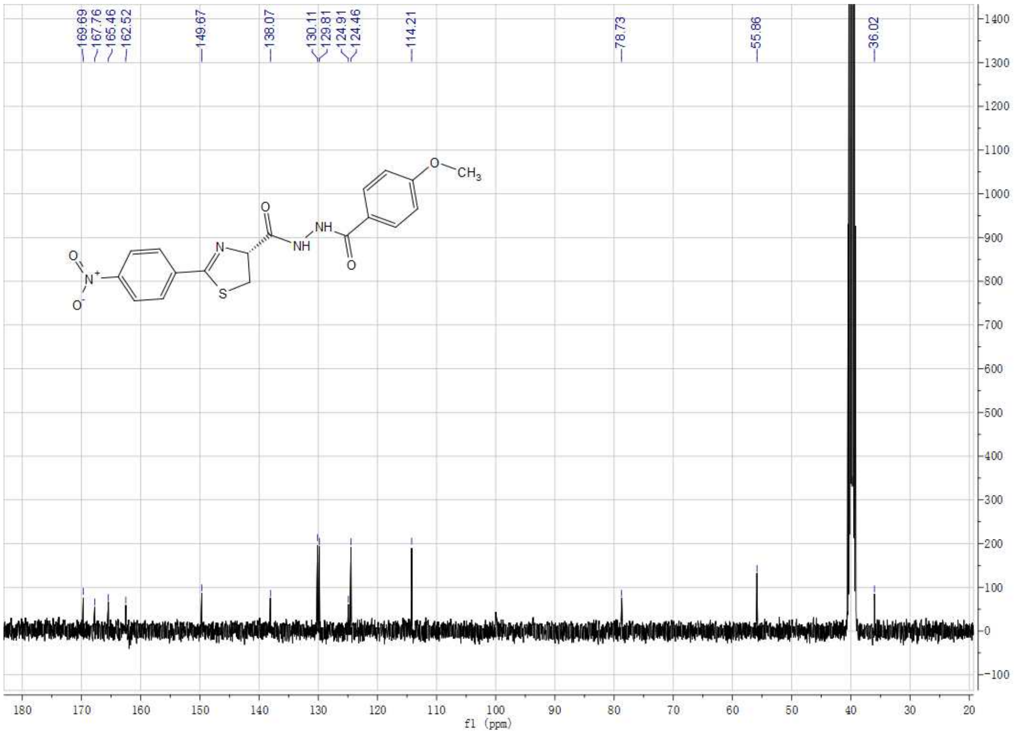

Compound II-4

^{1}H NMR

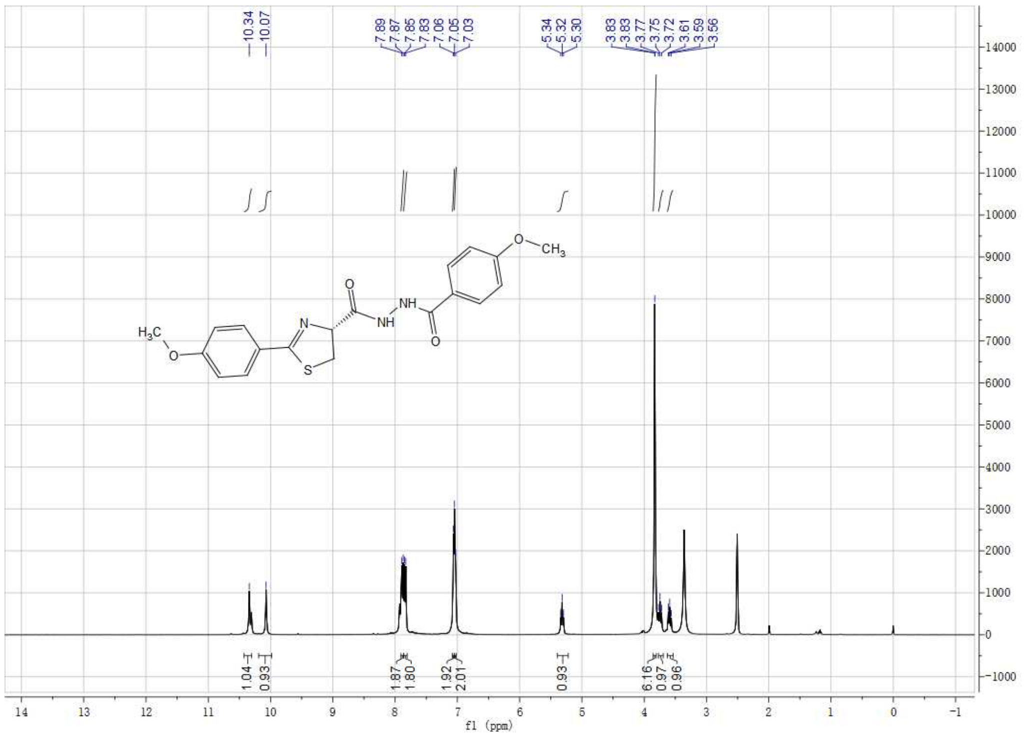

^{13}C NMR

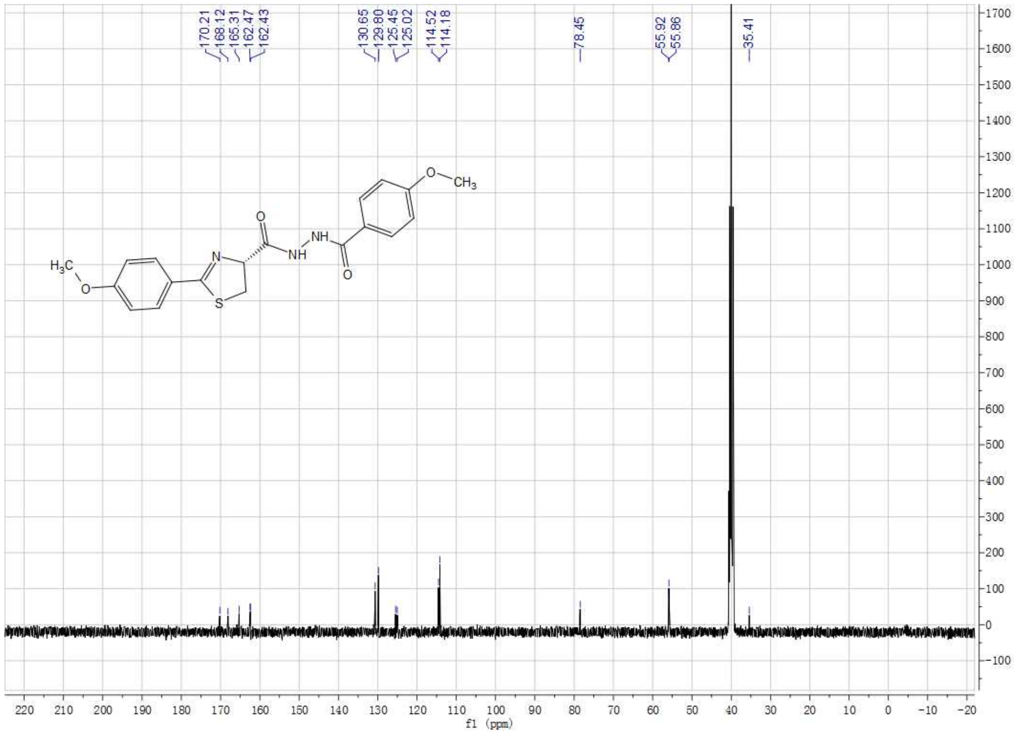

Compound II-5

^{1}H NMR

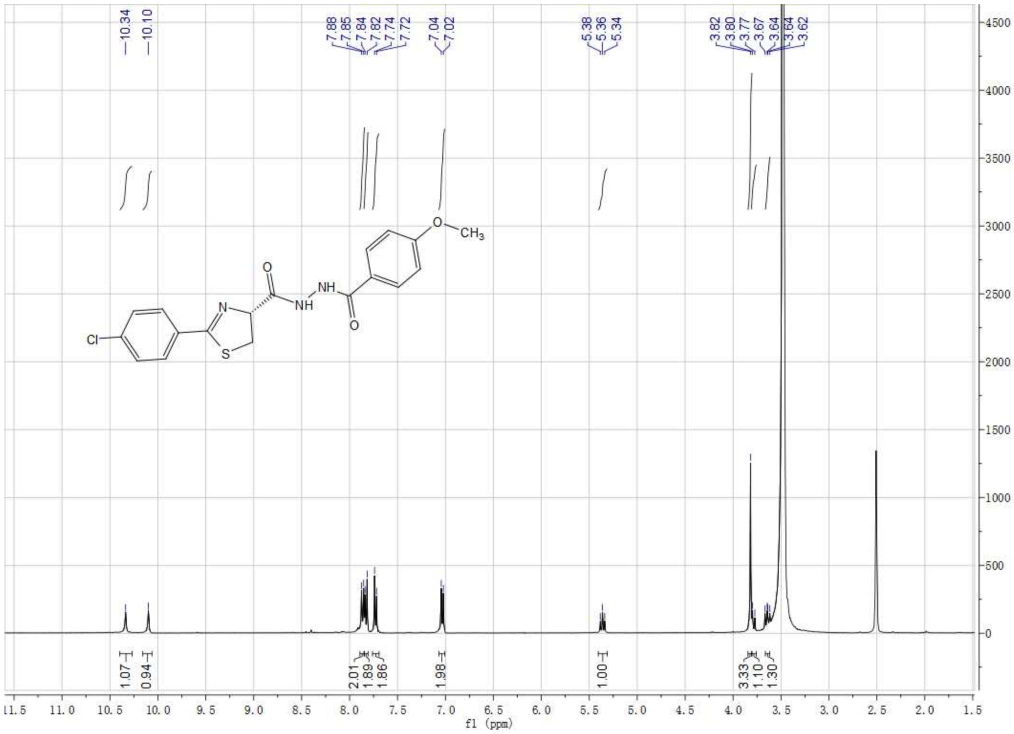

^{13}C NMR

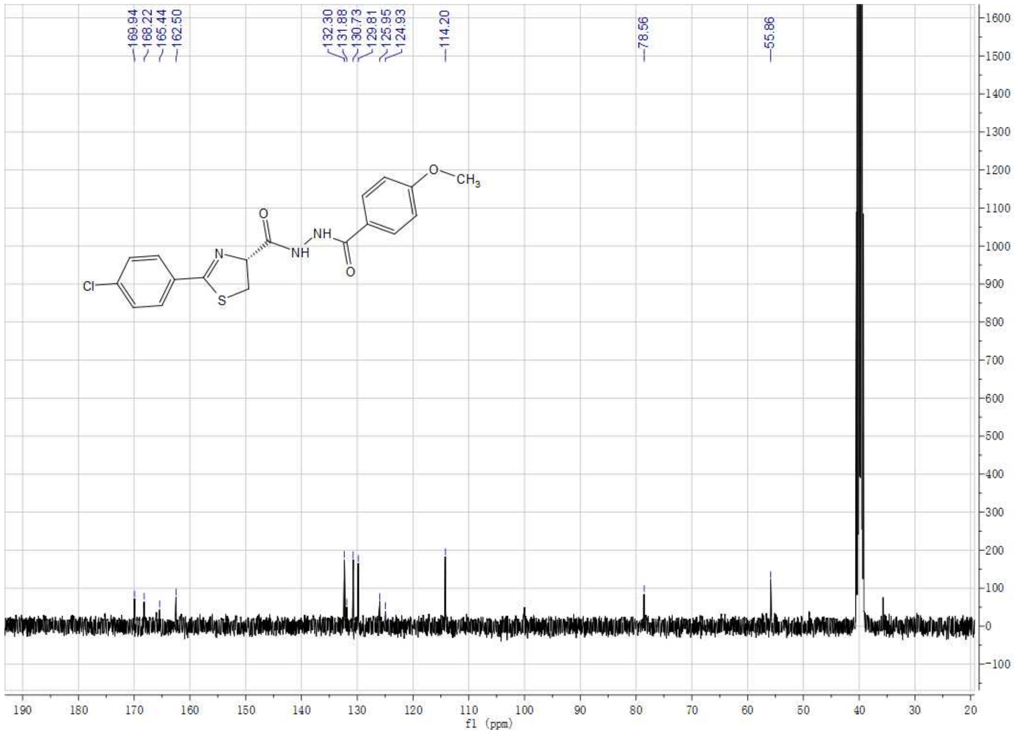

Compound III-1

^{1}H NMR

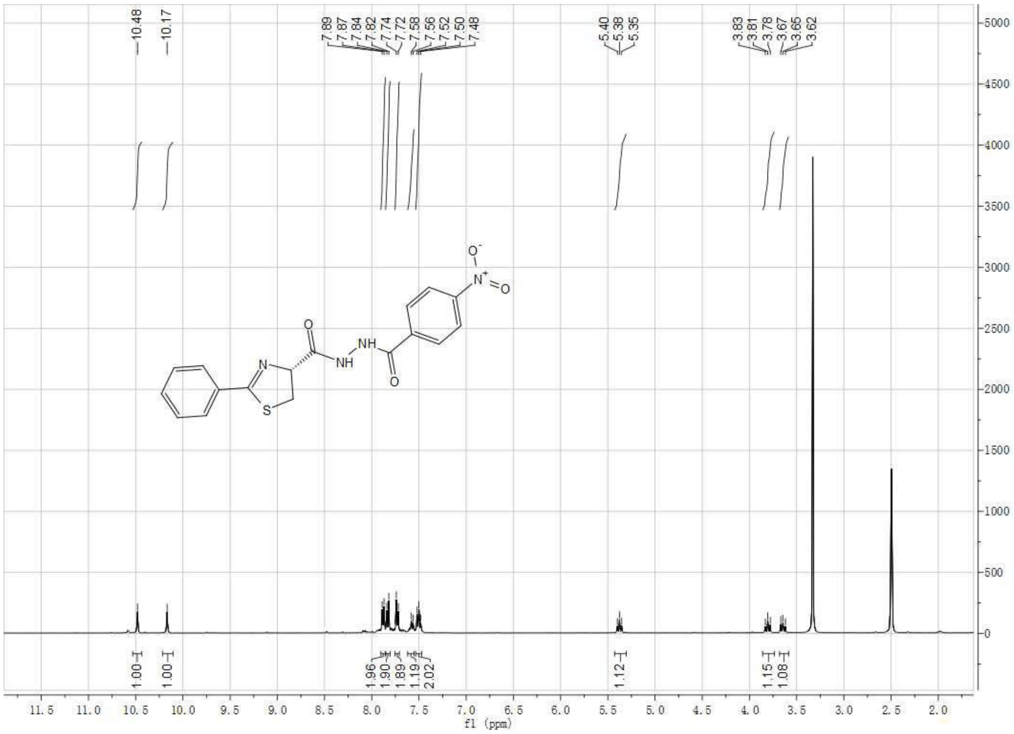

^{13}C NMR

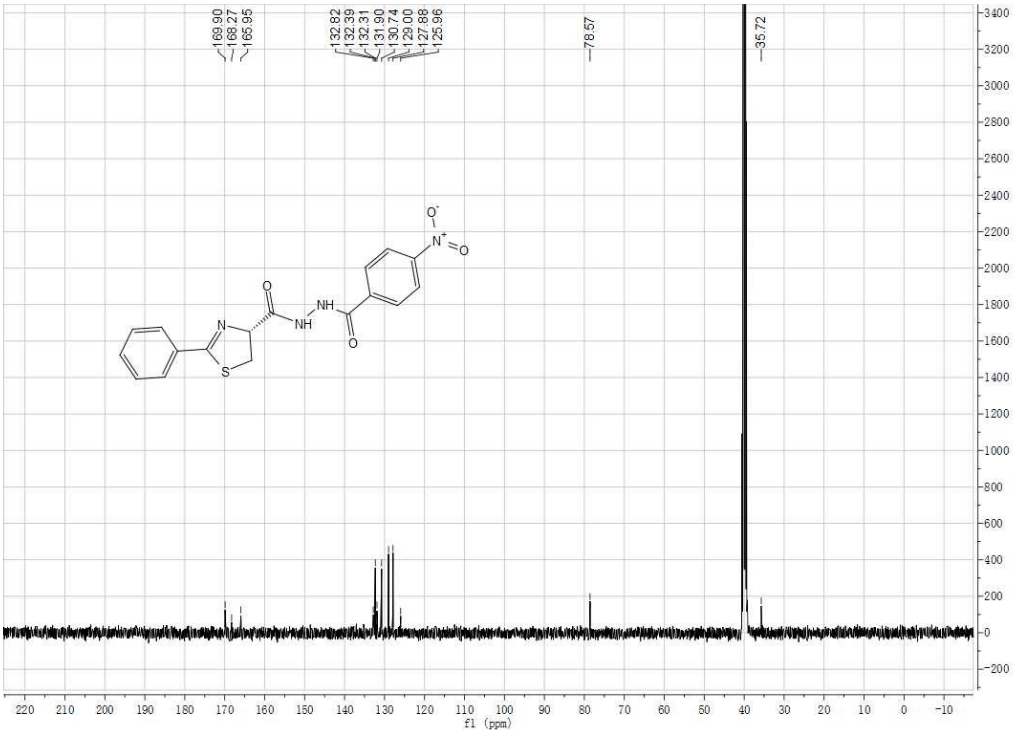

Compound III-2

^{1}H NMR

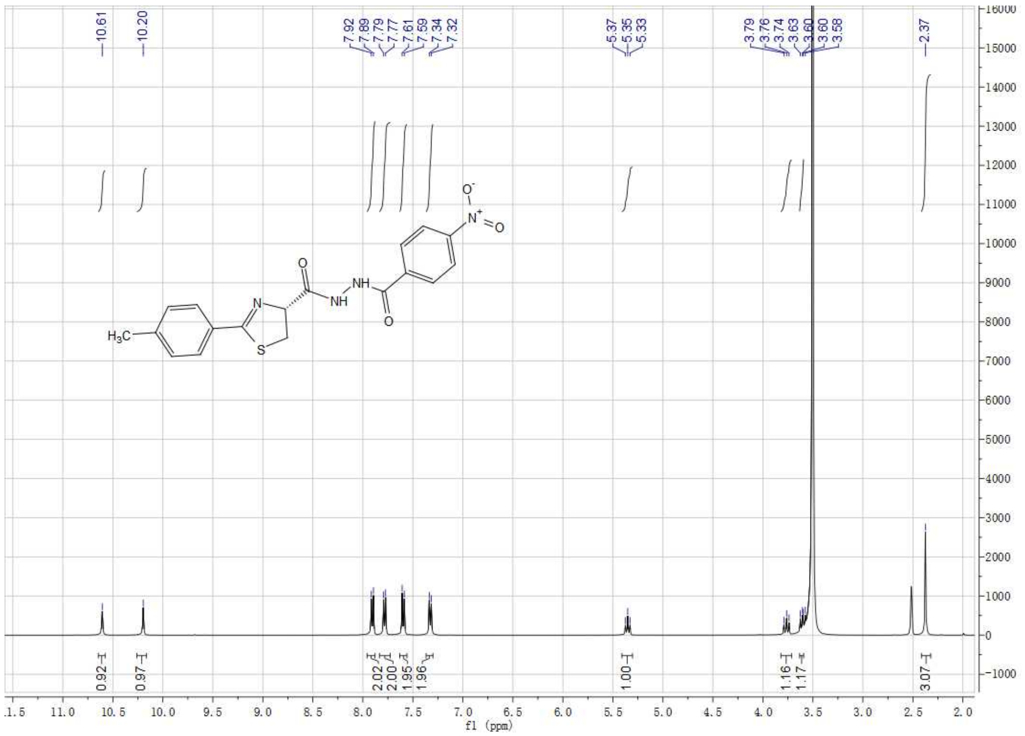

^{13}C NMR

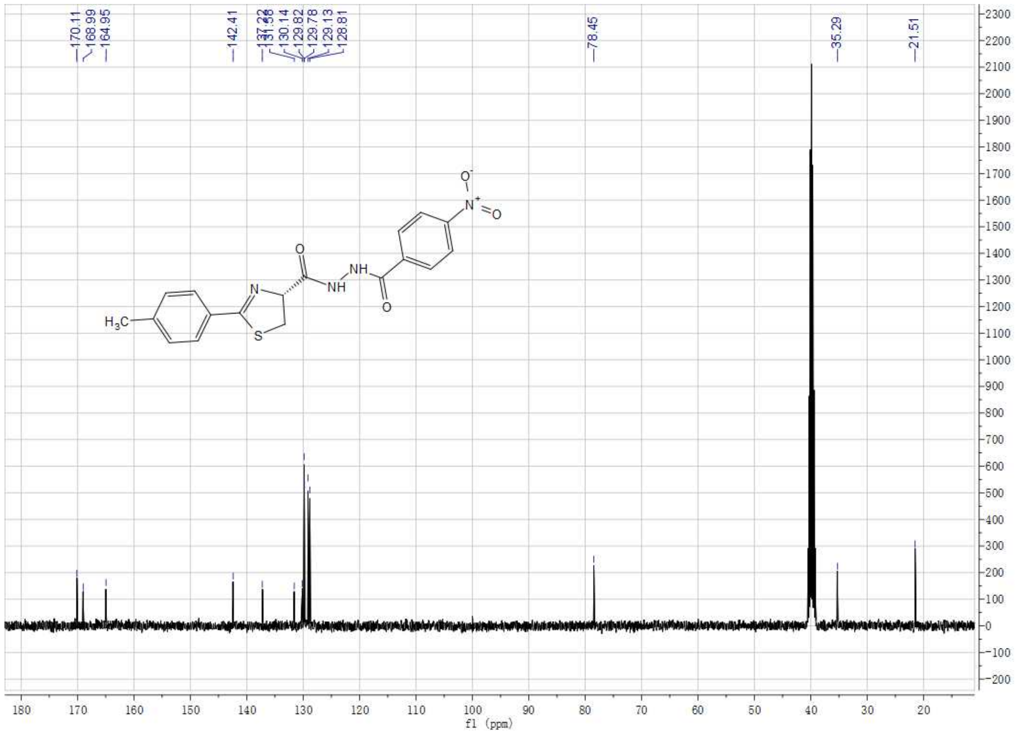

Compound III-3

^{1}H NMR

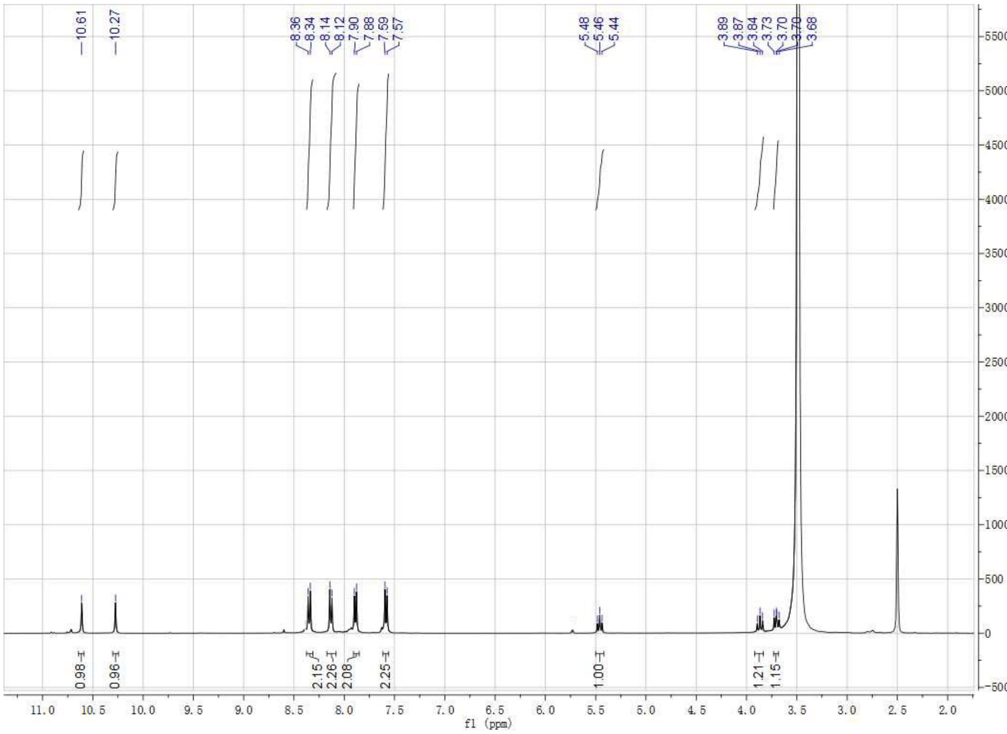

^{13}C NMR

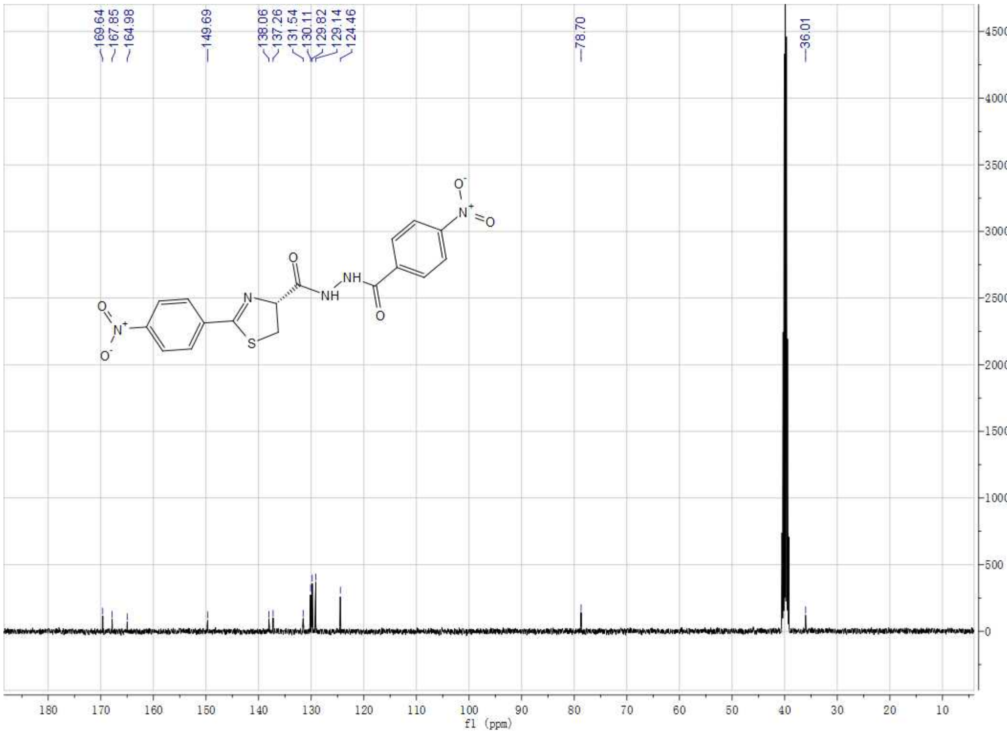

Compound III-4

^{1}H NMR

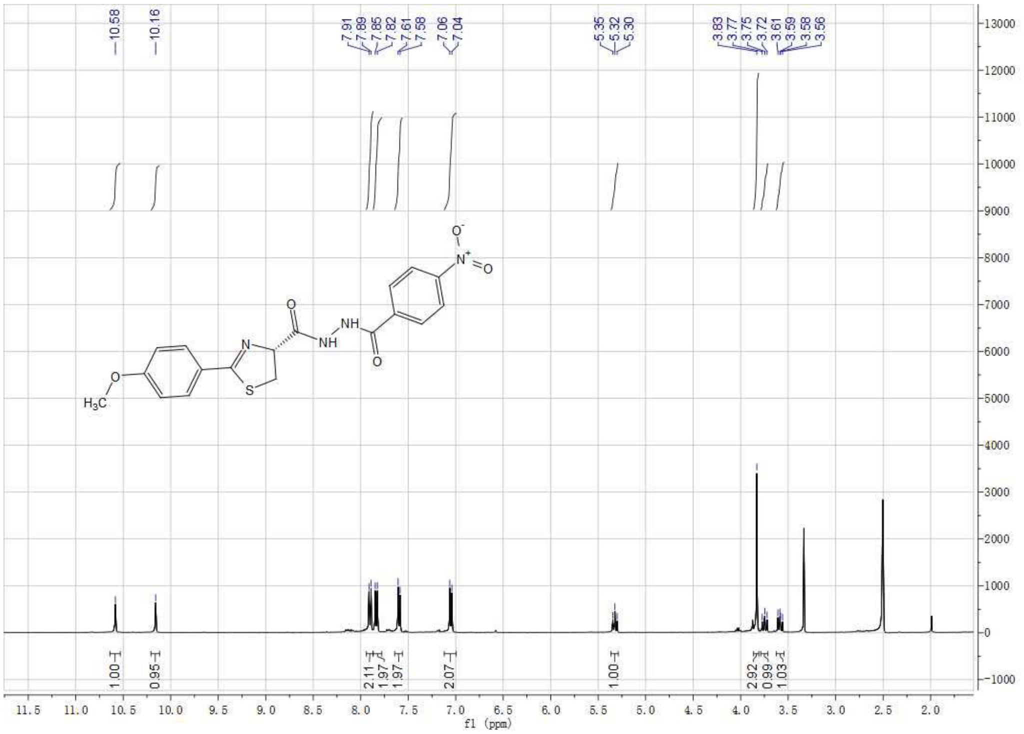

^{13}C NMR

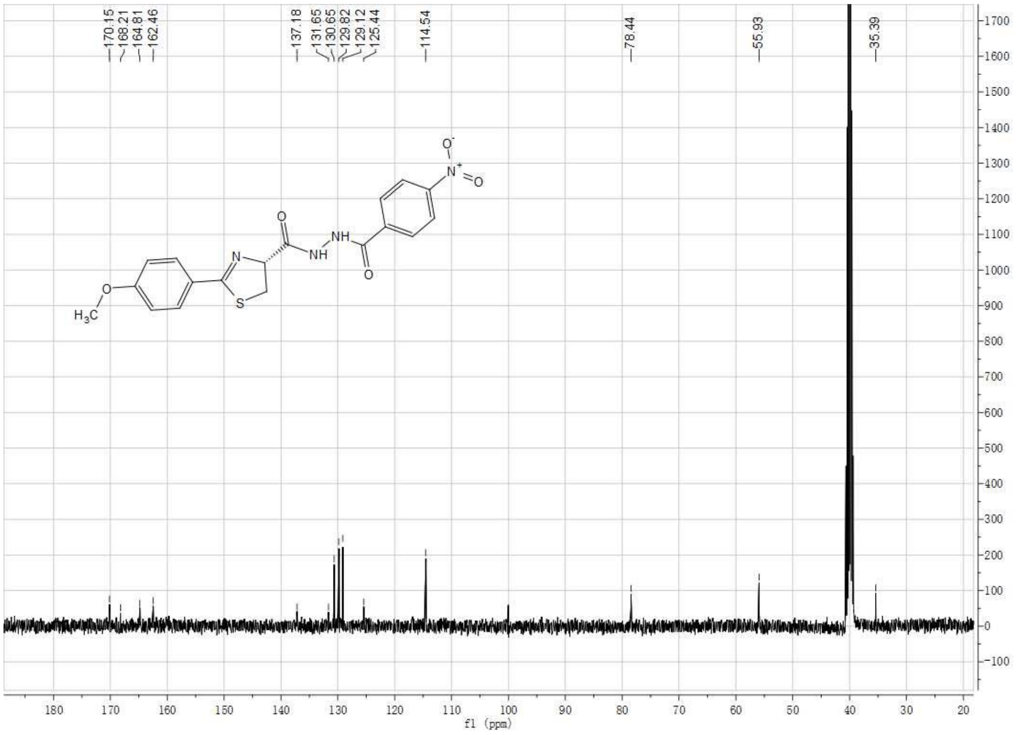

Compound III-5

^{1}H NMR

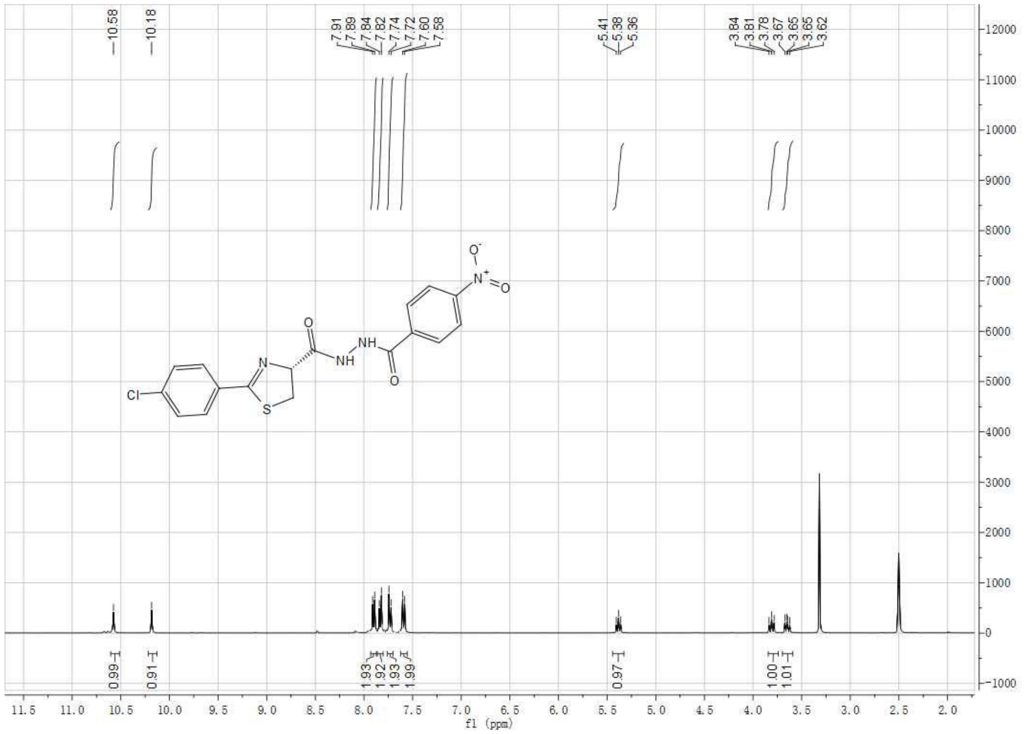

^{13}C NMR

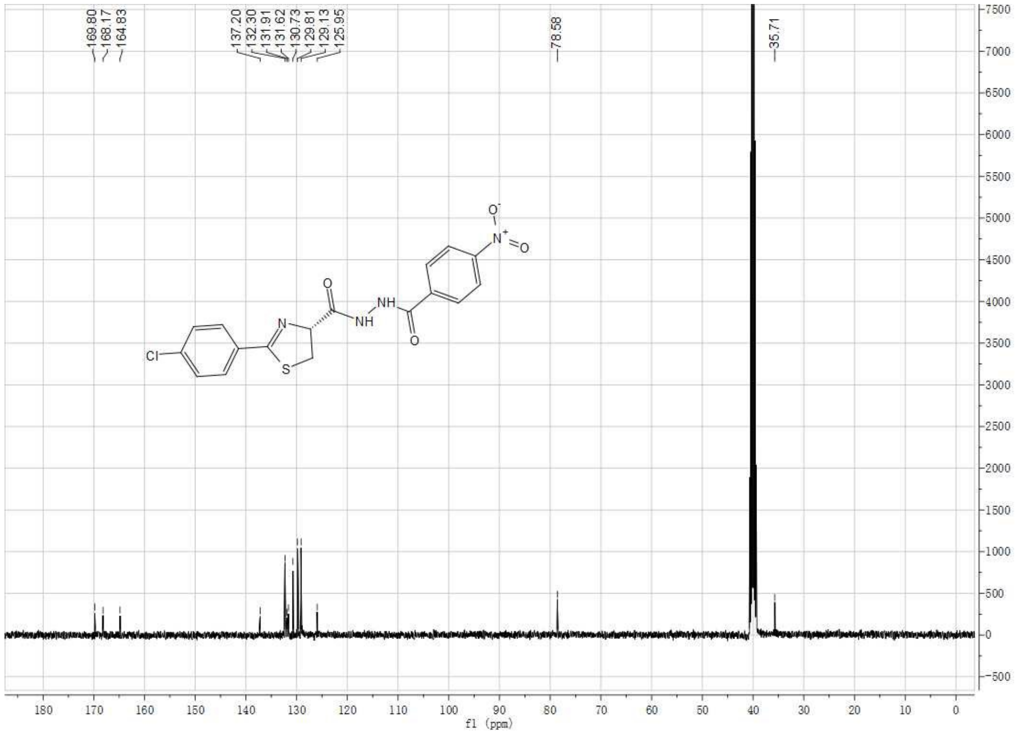

Supplement: Supplementary file 1 [file molecules-24-04440-s001.pdf]
